# Mobility in China, 2020: a tale of four phases

**DOI:** 10.1093/nsr/nwab148

**Published:** 2021-08-16

**Authors:** Suoyi Tan, Shengjie Lai, Fan Fang, Ziqiang Cao, Bin Sai, Bing Song, Bitao Dai, Shuhui Guo, Chuchu Liu, Mengsi Cai, Tong Wang, Mengning Wang, Jiaxu Li, Saran Chen, Shuo Qin, Jessica R Floyd, Zhidong Cao, Jing Tan, Xin Sun, Tao Zhou, Wei Zhang, Andrew J Tatem, Petter Holme, Xiaohong Chen, Xin Lu

**Affiliations:** College of Systems Engineering, National University of Defense Technology, Changsha 410073, China; WorldPop, School of Geography and Environmental Science, University of Southampton, Southampton SO17 1BJ, UK; College of Systems Engineering, National University of Defense Technology, Changsha 410073, China; College of Systems Engineering, National University of Defense Technology, Changsha 410073, China; College of Systems Engineering, National University of Defense Technology, Changsha 410073, China; College of Systems Engineering, National University of Defense Technology, Changsha 410073, China; College of Systems Engineering, National University of Defense Technology, Changsha 410073, China; College of Systems Engineering, National University of Defense Technology, Changsha 410073, China; College of Systems Engineering, National University of Defense Technology, Changsha 410073, China; College of Systems Engineering, National University of Defense Technology, Changsha 410073, China; College of Systems Engineering, National University of Defense Technology, Changsha 410073, China; College of Systems Engineering, National University of Defense Technology, Changsha 410073, China; College of Systems Engineering, National University of Defense Technology, Changsha 410073, China; School of Mathematics and Big Data, Foshan University, Foshan 510000, China; State Key Laboratory on Blind Signal Processing, Chengdu 610041, China; WorldPop, School of Geography and Environmental Science, University of Southampton, Southampton SO17 1BJ, UK; State Key Laboratory of Management and Control for Complex Systems, Institute of Automation, Chinese Academy of Sciences, Beijing 100190, China; Chinese Evidence-Based Medicine Center, National Clinical Research Center for Geriatrics, West China Hospital, Sichuan University, Chengdu 610041, China; Chinese Evidence-Based Medicine Center, National Clinical Research Center for Geriatrics, West China Hospital, Sichuan University, Chengdu 610041, China; Big Data Research Center, University of Electronic Science and Technology of China, Chengdu 611713, China; West China Biomedical Big Data Center, West China Hospital, Sichuan University, Chengdu 610047, China; WorldPop, School of Geography and Environmental Science, University of Southampton, Southampton SO17 1BJ, UK; Tokyo Tech World Hub Research Initiative, Institute of Innovative Research, Tokyo Institute of Technology, Tokyo 226-8503, Japan; School of Business, Central South University, Changsha 410083, China; Institute of Big Data and Internet Innovations, Hunan University of Technology and Business, Changsha 410205, China; College of Systems Engineering, National University of Defense Technology, Changsha 410073, China

**Keywords:** human mobility, travel restrictions, COVID-19, mobile phone data, behavioral response

## Abstract

2020 was an unprecedented year, with rapid and drastic changes in human mobility due to the COVID-19 pandemic. To understand the variation in commuting patterns among the Chinese population across stable and unstable periods, we used nationwide mobility data from 318 million mobile phone users in China to examine the extreme fluctuations of population movements in 2020, ranging from the Lunar New Year travel season (*chunyun*), to the exceptional calm of COVID-19 lockdown, and then to the recovery period. We observed that cross-city movements, which increased substantially in *chunyun* and then dropped sharply during the lockdown, are primarily dependent on travel distance and the socio-economic development of cities. Following the Lunar New Year holiday, national mobility remained low until mid-February, and COVID-19 interventions delayed more than 72.89 million people returning to large cities. Mobility network analysis revealed clusters of highly connected cities, conforming to the social-economic division of urban agglomerations in China. While the mass migration back to large cities was delayed, smaller cities connected more densely to form new clusters. During the recovery period after travel restrictions were lifted, the netflows of over 55% city pairs reversed in direction compared to before the lockdown. These findings offer the most comprehensive picture of Chinese mobility at fine resolution across various scenarios in China and are of critical importance for decision making regarding future public-health-emergency response, transportation planning and regional economic development, among others.

## Teaser

This article gives an overview of commuting patterns in China in 2020, offering the most comprehensive understanding on national mobility at fine resolution across various scenarios in China.

## INTRODUCTION

Understanding human mobility is fundamental to societies, with applications such as urban planning [1], traffic programming [2], epidemic modeling and control [3], and regional economic development at different geographic scales [4,5]. Data for human mobility studies can be extracted from census data or surveys [6,7], mobile phone data [8,9] (usually individual trajectories or aggregated population flows collected from call detail records, CDRs) or the Global Positioning System (GPS) data obtained from different devices [10,11]. Models [12–15] of human mobility demonstrate that human population movements are far from random, possessing a high degree of regularity [16] and predictability [17,18] at multiple spatio-temporal scales [19–22].

Mobility patterns change with the occurrence of major holidays [23] and the emergence of epidemics [24,25]. In early 2020 in China, these two factors coincided: on one side, the outbreak of the severe acute respiratory syndrome coronavirus 2 (SARS-CoV-2) was dramatically expedited by the high levels of domestic and international human mobility in this modern world [26,27]. On the other, the unfolding of the virus and interventions put in place might have significantly modified human behavior and movements [28]. Complex variations in mobility behavior cause standard models of human mobility to become less predictive. Therefore, a systematic understanding of the changes in human mobility across space and time during the COVID-19 pandemic is critical for assessing the risk of infectious disease transmission and the effectiveness of non-pharmaceutical interventions (NPIs) [29,30], thereby tailoring precise intervention strategies for future waves and pandemics [31,32].

The worldwide implementation of travel restrictions, event postponements, curfews, quarantines and physical distancing policies have resulted in a substantial decrease in population flow [33,34]. These restrictions have been proven to be one of the most effective measures for significantly reducing the transmission rate of COVID-19 in the absence of an effective vaccine [35,36]. The acceleration of COVID-19 transmission in China coincided with the annual *chunyun*, for which a population migration of nearly three billion trips around the Lunar New Year was recorded in 2019. Since the Wuhan travel ban on 23 January 2020, China has implemented stringent COVID-19 containment measures [37,38], and together with the massive efforts of residents in China to contain the disease, China has, to date, been considered to have implemented one of the most successful national strategies for COVID-19 suppression.

By analyzing aggregated, non-personally identifiable human movement data recorded before, during and after the first wave of the COVID-19 outbreak in China, we revealed the changing spatio-temporal features of mobility and examined the impact of China's COVID-19 containment policies on domestic travel in 2020. Specifically, we studied the changes of population mobility patterns during the *chunyun*, epidemic and intervention period—including aggregated flows, travel distance and community evolution—across 366 prefectures in Chinese mainland. The analysis of these patterns facilitates a deeper understanding of changes in human behavior [39], which will inform efforts to fight future waves of the pandemic, tailor control strategies and manage population movements to prevent the spread of new variants of concern in resurgences, as well as provide evidence for transportation planning and regional economic development [40,41].

## RESULTS

### An overview of population flows in China

**Figure 1.**
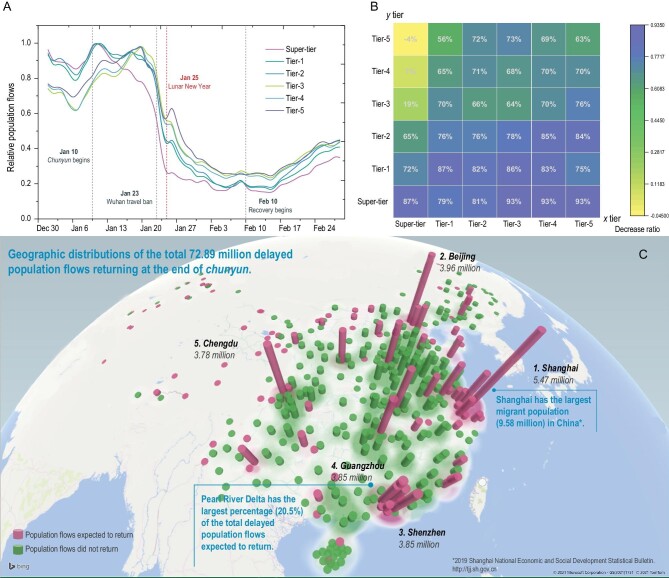
Temporal patterns of population flow in China in early 2020. (A) Aggregated population flows for all prefectures from 1 January to 29 February 2020. Each curve represents changes in flows for each prefecture tier over 60 days. Data were normalized by the maximum flow value in each tier. (B) The mobility decrease ratio before (from 17 to 23 January, the week prior to the implementation of lockdown measures) and after (from 1 to 7 February, the week after the Lunar New Year holiday) Wuhan travel ban. Each cell (*x* tier, *y* tier) represents the ratio for flow from cities in tier *y* to tier *x*. (C) The geographic distribution of the delayed population flows due to travel restrictions. These flows were expected to return at the end of *chunyun*.

A substantial increase in population flow was observed a few days before the start of the *chunyun* migration (Fig. 1A). Average cross-city daily movements increased from 107.06 million in normal times to 125.83 million for *chunyun*, from 10 to 23 January. The flow was followed by a sharp drop beginning on 23 January, when lockdown measures were implemented in Wuhan, with all public transport, including buses, railways, flights and ferry services, being suspended. With travel restrictions immediately adopted in other provinces following the Wuhan travel ban, this reduction was sustained for almost three weeks in China: the population flow reached the minimum level (29.15 million) on 15 February, dropping to nearly a quarter of the population flow of 22 January (124.87 million). From 24 January to 29 February, there were ∼45.05 million cross-city movements each day, on average, which was less than half of that on 22 January. After returning to work, the population flow did not return to previous levels, even at the end of the study period.

Compared with one week earlier, when lockdown measures were implemented, the population flow decreased by over 70% in the week after the Lunar New Year holiday. The average decrease ratios for population outflow were typically larger in high-tier prefectures (the concept of prefecture tier is a hierarchical classification of Chinese prefectures that describes the prefecture's relative level of development, see details in Methods). Significantly, population outflow from the super-tier prefectures (mean decrease ratio = 0.876) declined much faster than the other prefectures’ outflow (Fig. 1B). In contrast, population inflow to the super-tier prefectures from the fourth-tier and fifth-tier prefectures remained nearly unchanged (mean decrease ratio = 0.07 and –0.04, respectively). Assuming that the excessive population flow during *chunyun* would have otherwise returned to its originating cities, the travel restrictions delayed more than 72.89 million people returning by the end of *chunyun* (18 February), mainly for work and education purposes. For Beijing, Shanghai, Guangzhou and Shenzhen, this number holds for 17.14 million (Fig. 1C). High-tier prefectures were the leading destinations of those delayed population flows, while the low-tier prefectures around the neighboring central prefectures retained a large fraction of population flows. Changes in human movements were partially driven by NPIs [42] such as lockdown measures, event cancellations, and university and business closures. Additionally, many citizens spontaneously obeyed social distancing measures, staying at home for as long as possible. As a result, a consistent decline in population movement effectively contained the spread of COVID-19 in China [43].

The directions of population flow between pairs of cities changed significantly among prefecture tiers. There was a substantial drop in inflows for the highest three prefecture tiers starting on 10 January 2020, the start of *chunyun*. The outflows of these higher tiers were much higher than their inflows. However, the lowest three prefecture tiers exhibited increases in inflows and outflows, which peaked on 20 January. The outflows of these lower tiers were smaller than their inflows (Fig. S1 in the online supplementary data). Besides, we found that neither the population size nor distance of the city to Wuhan was correlated to the decrease ratio of population movements, indicating that the societal response and implementation of containment measures to the outbreak of COVID-19 is universal in China, regardless of the proximity or social-economic development of the location (Fig. S2).

### Mobility network variation across periods

**Figure 2.**
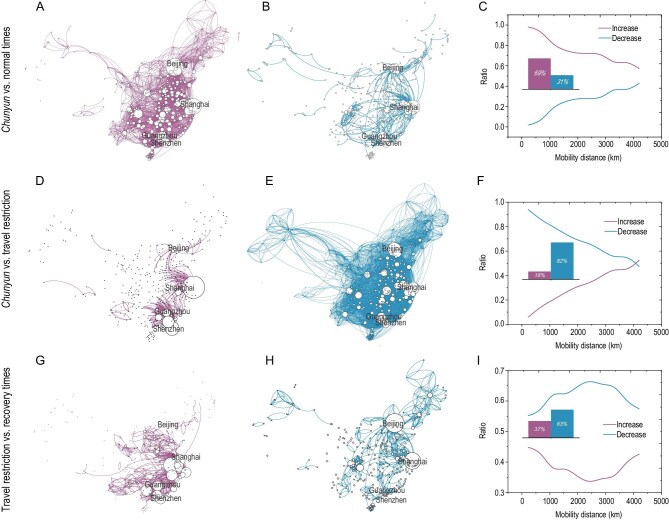
A comparison of spatial patterns of mobility across four periods. The mobility network is visualized with links that decrease/increase by more than 200 in average daily flows. (A, D and G) Links that exhibited increases in population flow in comparison with the previous period. The size of the node is proportional to total outflows for (A), and total inflows for (D and G). (B, E and H) Links that exhibited decreases in population flow in comparison with the previous period. The size of the node is proportional to total inflows for (B and H), and total outflows for (E). (C, F and I) The association of mobility distance with the ratio of links with increased or decreased travel. Each bar shows the ratio of links with increased or decreased travel, and each curve shows the variation tendency.

Compared with normal times, population flows notably increased during the *chunyun* migration period, and over 68% of all pairs (directed) of cities had an increase in population flows. Along the national mobility network, the average daily flow of 4588 links (directed pairs of cities) increased by >200, mainly due to outflows from super-tier and first-tier prefectures with large populations and high GDPs to lower-tier prefectures (Fig. 2A). Conversely, inflow to these higher-tier prefectures dropped simultaneously (Fig. 2B).

During the travel restriction period, few migrant workers [44] returned to workplaces located in the most developed regions in China: the Yangtze River Delta (YRD) and the Pearl River Delta (PRD) Greater Bay Area. There were only 216 links with an increase in average daily flow of >200 during this period (Fig. 2D). These regions contain many of the most high-tech, capital-intensive manufacturing industries in China, which provide many jobs and competitive salaries. Return-to-work migration contributed to the increase in population flows from 10 to 29 February (Fig. 2G). During the COVID-19 epidemic, human movements decreased due to travel restrictions, such as lockdown measures and stay-at-home orders. All city pairs exhibited a massive 82.46% decline in population flows. The

average daily flows of 9004 links were reduced by >200 (Fig. 2E).

Even during the period when people were returning to work, population flows had not yet returned to their prior levels. Over 62.64% of all pairs of cities had a decrease in population flow. The average daily flows of 1795 links reduced by more than 200, especially for inflows to Beijing and Shanghai, the capital and financial center, which implemented strict intervention measures, including more stringent controls on the movement of residents and vehicles, mandatory temperature checks and compulsory mask-wearing in public (Fig. 2H).

The initial increase and subsequent dramatic decrease in human movements clearly showed how *chunyun* and the following COVID-19 epidemic affected human mobility patterns. During the *chunyun* migration period (vs. normal times), the flow decrease ratio was positively correlated with travel distance, while the flow increase ratio was negatively correlated with travel distance (Fig. 2C). During the epidemic (vs. the *chunyun* migration period), the increase ratio was positively correlated with travel distance, while the decrease ratio was highly negatively correlated with travel distance (Fig. 2F), suggesting that shorter distances saw a much higher decrease in flows than longer distances during this time. Immediately following the lockdown of Wuhan and travel restrictions across Chinese mainland, the decrease ratio was more dramatic than the increase ratio during the last two periods (24 January to 29 February). When the government called for reopening of the economy and schools, increased population flows were observed over both short and long distances. In contrast, travels over medium distances continued to decrease (Fig. 2I).

### Travel distance analysis

**Figure 3.**
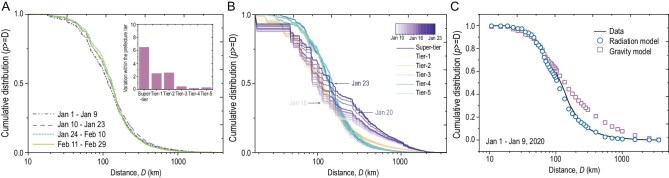
Overall population movements. (A) Plots of cumulative probability distribution against daily travel distance (log) across all four periods. The subgraph shows the variability of movement within prefecture tiers across all four periods. The cumulative probability distribution of daily travel distances is denoted by *p*, and the mean distribution over all 60 days is denoted by$\bar{p}$. For each day *k*, we calculate the proximity of ${p_k}$ to ${\bar{p}_k}$ to express the variability of movement, and then we arrive at the sum of 60 days’ variation. (B) Plots of the cumulative probability distribution of movements grouped by prefecture tiers against distance (log) for *chunyun* (10–23 January). Each line represents the probability distribution per day. (C) Plots of travel distance distributions produced by the gravity and radiation models, compared with real data during normal times.

Figure 3A shows the cumulative distribution of travel distances over all four periods. Short-distance travel occurred more frequently during normal times, while long-distance travel occurred more frequently during the *chunyun* migration period. In this period, movements across >100 km increased by 5%; however, people were more likely to stay at home or travel shorter distances following the COVID-19 outbreak due to travel restrictions. This evidence suggests that long-distance travel decreased during the last two periods. Detailed summary statistics concerning travel distances are provided in Table S1 in the online supplementary data. Unsurprisingly, the mobility patterns for the *chunyun* migration period coincided with that of the

recovery period, as migrant workers might have traveled home when the *chunyun* began and then returned to work, producing a similar but reversed net population flow.

To better understand the mass increase in migration during *chunyun*, we plotted cumulative probability distributions against distance with the dates of *chunyun*. With each day, travel distances of the super-tier prefectures increased progressively towards Lunar New Year's Eve. An increase in average daily travel distances was also found in the first-tier and second-tier prefectures. However, travel distance distributions remained nearly the same for prefectures at the lower (third, fourth and fifth) tiers, as their mobility patterns had stabilized considerably (Fig. 3B). Additionally, mobility patterns differed substantially between tier groups but showed strong consistency within each tier group over all 60 days (Fig. 3A, inset). Although there are many models of human mobility (e.g. the radiation model [12] and the gravity model [13]), according to the goodness of fit for the mobility models evaluated by the root mean square error (RMSE), the radiation model outperforms the gravity model, as well as a series of other mobility models (Fig. S3 and Table S2), in predicting the population flows for all four periods (Fig. 3C). However, there is still a significant prediction discrepancy for population flows within short distances (e.g. ≤150 km). Therefore, we do not have a well-understood model to account for the mobility pattern wherein *chunyun*, epidemic and lockdown are intertwined.

### Quantifying the backflow effect

**Figure 4.**
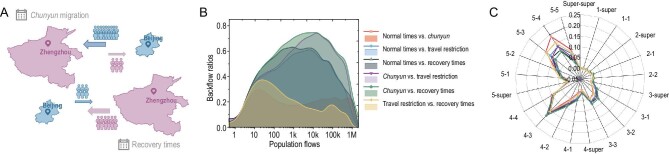
Formalization of backflow variation modes in human mobility patterns. (A) An illustration of the hypothesized backflow pathways between two prefectures. (B) Plots of backflow ratios (the fraction between backflow links and total links occurring across all pairs of cities at the two compared periods) versus aggregate population flows (the total flow during two compared periods on the same link). Pairwise comparisons were made for every two periods. (C) Backflow ratios for all pairs of prefecture tiers.

Since *chunyun* is a temporary relocation that occurs over the Lunar New Year holiday, people might have traveled when *chunyun* began and then traveled back to their prior locations afterward. Throughout the process, the return trips roughly matched the outward trips. We refer to this as the backflow effect (Fig. 4A). For example, thousands of migrant workers traveled to their homes during *chunyun* and then returned to their jobs during the recovery period, reversing the direction of netflows along the mobility network. In fact, over 55% of all links exhibited reversed netflow between *chunyun* and the recovery time. We developed a mathematical model to explore the backflow phenomenon across all prefectures (see Methods). The ratio of links with reversed netflow initially increased markedly with small population flows and then declined with large population flows (Fig. 4B). A detailed investigation of the decreasing trend revealed that large population flows usually exist in neighboring prefectures that are geographically connected (Table S3), such as Guangzhou and Foshan (distance = 30 km; 400 145 movements per day) or Beijing and Langfang (distance = 40 km; 70 148 movements per day). This mobility pattern is a kind of daily commuting pattern rather than backflow. Furthermore, the backflow phenomenon typically occurred between lower-tier prefectures, e.g. third-tier, fourth-tier and fifth-tier prefectures (Fig. 4C). The distribution of reverse movements that occurred between every two periods are shown in Fig. S4.

### Evaluation of community dynamics

**Figure 5.**
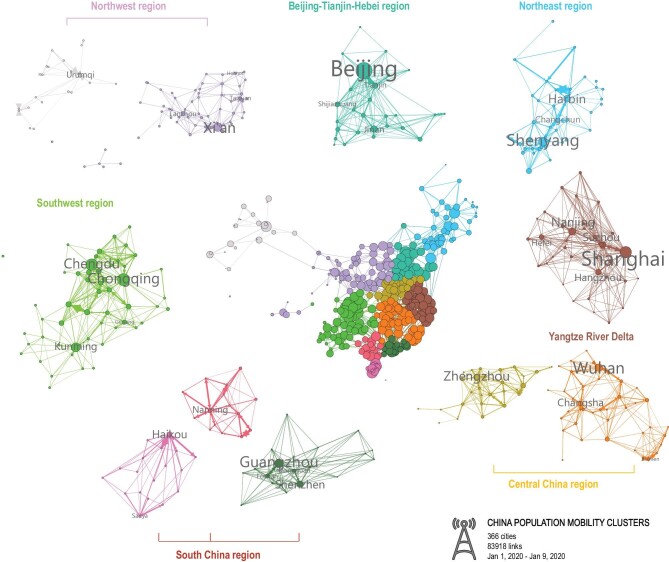
The community structure of all prefectures during normal times in Chinese mainland. Nodes of the same color belong to the same community, as detected by the Louvain algorithm. Link weights indicate the directed average daily number of trips between the two connected nodes, which shows the strength of the interaction between two prefectures. The community structure network is visualized with links weighted over 10 000.

Crowd movement reveals socio-economic and cultural interactions among prefectures, which form city clusters. We used the Louvain algorithm [45] to detect community dynamics of the national temporal mobility network and found that the formation of communities across China mainly depends on regions of urbanization and economic development. For example, Beijing, Tianjin, Jinan and Shijiazhuang form the Beijing-Tianjin-Hebei region

(Jing-Jin-Ji); Guangzhou, Shenzhen, Foshan and Dongguan form the PRD; and Shanghai, Nanjing, Hangzhou and Hefei form the YRD. These regions have been identified as the three world-class city clusters in China (Fig. 5 and Table S4).

**Figure 6.**
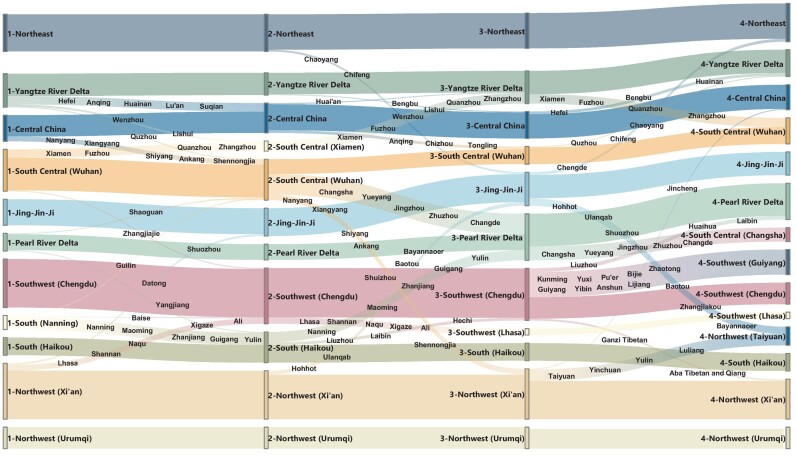
Alluvial diagram for mapping community dynamics in the mobility network. The height of a streamline between two stages is proportional to the number of movements that migrate between two communities.

The observed community changes during *chunyun* were mainly due to laborers returning to hometowns from cities principally located in the wealthy coastal regions that are far from their home regions. For example, many prefectures from Anhui Province, such as Hefei, Anqing and Lu’an, left the community located in the YRD region, and merged into the neighboring community, which is located more geographically inland. During the period from *chunyun* to the travel restrictions, COVID-19 impacted economic activities and restricted population movements across the country. For example, most prefectures in Hunan Province, which were closely connected with Hubei Province, left the community centered around Wuhan, and merged into the community centered around Guangzhou. The number of communities was 11 before the lockdown, and increased to 14 during the recovery period (Tables S5–S7 in the online supplementary data). The three new communities are centered around Changsha (including 15 neighboring prefectures), Taiyuan (including 20 neighboring prefectures) and Guiyang (including 27 neighboring prefectures). The subdivision of communities into smaller communities revealed that stronger local interactions were generated in response to the stay-at-home order and other legally enforced mobility restrictions (Fig. 6).

In all four periods, most high-tier prefectures were more likely to be located in the same region (usually centered around two or three neighboring central prefectures) than low-tier prefectures before and after the COVID-19 outbreak. Higher-tier prefectures have integrated overall strengths, which amplified their influence and radiation effect and promoted the socio-economic development of regional integration, thus creating more opportunities for them to draw substantial population inbound from lower-tier prefectures. Approximately one-third of the prefectures (126 out of 366) exhibited changes in their communities. Of these prefectures, over 90% belonged to the lowest three prefecture tiers; only two of these prefectures belonged to the first-tier (Changsha, Hefei). Twenty prefectures ultimately returned to their initial communities. The south-central cluster (Wuhan, Changsha and Xiamen are the central prefectures) was seriously affected by the Wuhan travel ban. Changsha, Xiamen, as well as their neighboring prefectures, moved to PRD and YRD, respectively. The number of prefectures in the south-central community declined from 45 to 16.

## DISCUSSION

Human mobility is the driving force of many social dynamics, including socio-economic development, disease transmission and forming of new fact-to-face interactions. Within the first two months of 2020, China experienced extreme fluctuations in the travel patterns of its population, ranging from the *chunyun* (maybe the busiest mass migration on Earth) to the exceptional calm of COVID-19 lockdown. The *chunyun* mass migration has influenced China's mobility levels, and the large-scale outbreak of COVID-19 that began in December 2019 has added some complications. Characterizing mobility at a large-scale and with high resolution is of critical importance to the understanding and policy making of everyday life. With data extracted from mobile phones at the national level, our study offers comprehensive analyses of bulk population movements in China, across distinct periods before and after the COVID-19 outbreak.

We found that variations between population flows were strongly correlated with travel distances across all four investigated periods. Daily movements changed as a function of distance much more remarkably in high-tier prefectures than in low-tier prefectures, and China's decline in mobility levels overall might have been due to both government interventions and personal behavioral changes. Interestingly, it appears that substantial reductions in mobility occurred continuously since the implementation of travel restrictions, with more than 72.98 million people delaying their return to cities until the end of *chunyun*. Our mathematical model for analyzing backflow mobility also revealed that this phenomenon mainly occurred between two prefectures belonging to low, proximal prefecture tiers. Despite large variations in mobility at the national scale, we found that clusters of prefectures formed by densely connected cities remained stable across the extreme periods of mass migration and travel restrictions.

The best public health strategy for minimizing serious outcomes in viral outbreaks is to control the viral spread at the earliest stage. A systematic understanding of the mobility patterns of populations and subsequent outcomes is clearly an important agenda item for urgent policy decisions. The present examination of mobility levels provided profound insight into how people move in response to emergencies, such as mass migration and widespread epidemics [46]. We believe the presentation of these results provides a state-of-the-art understanding of China's migration patterns during both stable and unstable scenarios. Additionally, as aggregated mobility data can provide near real-time information regarding changes in human mobility patterns, it can also be used to evaluate the effectiveness of government public health policies, such as travel restrictions and the integration of NPIs and vaccinations, thereby contributing to the fight against future waves of this pandemic and the threat of new variants. With accurate mobility information, the degree of strictness with which governments implement travel restrictions should be varied and adjusted from region to region over time. Since the low-tier prefectures retained a large fraction of their population flows by the end of *chunyun*, it was revealed that the control of in-province mobility is more important than mobility across provinces for those regions. When people resume work, specific guidelines for the authorities of high-tier prefectures may be beneficial in protecting inbound migrant workers. The patterns found in this study are valuable in assessing and revising existing lockdown measures, not only for the present, but also for when life resumes, without risking a major resurgence of this pandemic.

## METHODS

### Mobility data

Nationwide cellular signaling data (CSD) was used to track population flows throughout China at the prefecture (city) level. The data is recorded when users are making phone calls, sending messages, switching on/off their devices or switching towers. The temporal resolution is at a minimum of one record every 30 minutes because the CSD is recorded as long as there is an active or passive positioning data event. The spatial resolution was originally at the tower level, then aggregated to the prefecture-level, covering all prefectures in Chinese mainland. The population flow data is provided by one of the largest national mobile carriers in China, China Unicom, and is aggregated based on all users’ mobile phone activity records across the country, including geographic location. China Unicom had 318 million active users by the end of 2019 [47], about one quarter of all active mobile phone users in China (the other two operators are China Mobile and China Telecom). It is worth noting that, as all data were processed anonymously and aggregately, it is impossible for the authors to identify or filter users of certain groups. Thus, the population flow presented in this article provides a representative overview of the general population and cannot be analyzed for minority groups (see details in the online supplementary data).

The number of user trips in Chinese mainland (366 prefecture-level cities in total) from 1 January 2020 to 29 February 2020 were aggregated to generate a national-level population flow matrix. If the phone is on, the user's location will be recorded, whether users use it or not. To exclude a large number of users who only briefly pass through a prefecture, users who stay in a prefecture for less than half an hour were filtered out. The movement was recorded by the operator such that on each day, if a user was observed at locations A→B→C for more than 30 minutes, respectively, then A→B and B→C were counted.

The concept of prefecture tier is a convenient way to quickly describe the prefecture's relative level of development in Chinese mainland. The Rising Lab, a subsidiary of Yicai Media Group, has assessed the prefectural level for the sixth consecutive year, and the classification criteria includes five dimensions: concentration of commercial resources, the city as a hub, urban residential activity, lifestyle diversity and future potential. Beijing, Shanghai, Guangzhou and Shenzhen formed the super-tier. There were 15 prefectures in the first-tier, headed by Chengdu and Chongqing; 30 prefectures in the second-tier; 70 prefectures in the third-tier; 90 prefectures in the fourth-tier and 127 prefectures in the fifth-tier [48]. Details of the classification are presented in Tables S8–S12.

### Backflow model

For each day *k*, the population flow matrix is denoted by${A^k}$, where ${a_{ij}}$ and ${a_{ji}}$are the outflow and inflow from prefectures *i* to *j*, respectively. The unidirectional netflow matrix is denoted by ${E^k}$, where ${e_{ij}} = {a_{ij}} - {a_{ji}}$ and ${e_{ji}} = {a_{ji}} - {a_{ij}}$. Then, 60 unidirectional netflow matrices ${E^k}$can be derived from 60 days of population flow matrices ${A^k}$. A logical *xor* is used for the following symbolic expression:

(1) \begin{equation*} S_{ij}^{kl} = xor\! \left( {E_i^k,E_j^l} \right), \end{equation*}

where *k* and *l* are the *k*th and *l*th days, respectively. If ${e_{ij}} \in E_i^k$ and ${e_{ij}} \in E_j^l$ have the same signs, then $S_{ij}^{kl} = 0$. Otherwise, $S_{ij}^{kl} = {1}$, indicating opposite variation in population flows between prefectures *i* and *j* for the *k*th and *l*th days. To attain good statistics regarding the variation of population flow, this analysis was restricted to variations of flow >10 in both matrices ${A^k}$ and ${E^k}$. The reversed netflow direction is illustrated in Fig. 4A.

## DATA AVAILABILITY

Codes for the model simulations and data analysis are available upon request from S.Y.T. and X.L. The aggregated mobility matrices analyzed in this study can be requested from the corresponding author X.L.

## ETHICAL APPROVAL

This study was approved by the Ethics Review Board of West China Hospital, Sichuan University (2020-99).

## Supplementary Material

nwab148_Supplemental_File
