# Supplementary material for: Mobility in China, 2020: a tale of four phases
Source: Natl Sci Rev. 2021 Aug 16;8(11):nwab148. doi: 10.1093/nsr/nwab148 (PMC8645011; doi:10.1093/nsr/nwab148)
Supplement: nwab148_Supplemental_File [file nwab148_supplemental_file.docx]

Supplementary data for

**Mobility in China, 2020: a tale of four phases**

Suo-yi Tan^1†^, Shengjie Lai^2†^, Fan Fang^1†^, Ziqiang Cao^1^, Bin Sai^1^, Bing Song^1^, Bitao Dai^1^, Shuhui Guo^1^, Chuchu Liu^1^, Mengsi Cai^1^, Tong Wang^1^, Mengning Wang^1^, Jiaxu Li^1^, Saran Chen^3^, Shuo Qin^4^, Jessica R. Floyd^2^, Zhidong Cao^5^, Jing Tan^6^, Xin Sun^6^, Tao Zhou^7^, Wei Zhang^8^, Andrew J. Tatem^2*^, Petter Holme^9*^, Xiaohong Chen^10,11*^, and Xin Lu^1†*^

*Corresponding authors. Email: [andy.tatem@gmail.com](mailto:andy.tatem@gmail.com) (AT); [holme@cns.pi.titech.ac.jp](mailto:holme@cns.pi.titech.ac.jp) (PH); [csu_cxh@163.com](mailto:csu_cxh@163.com) (XC); [xin.lu@flowminder.org](mailto:xin.lu@flowminder.org) (XL).

**This PDF file includes the following:** Supplementary text about data description and analysis; Figs. S1–S9**;** Tables S1–S12.

Supplementary Text

**Data extrapolation and validation**

The mobile phone data used for this study was aggregated from cellular signaling data (CSD), which are collected by China Unicom with rich spatio-temporal information. The data contains real-time base-station-dependent location information from mobile phones, which is recorded when users are making phone calls, sending messages or switching on/off their devices. To enhance the extrapolation and representation of the population, user coverage, ratio of calls with other operators, as well as a variety of parameters extracted from the structure of users’ age, gender, etc., were combined and modelled using a machine learning approach by the operator, generating estimates on the number of migrating users from the whole network. The operator helped to extrapolate the data to all users of the entire network.

We have implemented a comprehensive validation analysis on both user coverage and data representativeness. It revealed that the mobile phone data-based extrapolation on population flow were in good agreement with GPS-based LBS (location-based services) data and official estimates on population flow during the study period. The study period coincided with the run-up to the annual *chunyun* mass migration and the COVID-19 epidemic. The 60 days covered by the study were then divided into four periods: normal times (January 1 to January 9), *chunyun* migration (January 10 to January 23), stringent travel restrictions (January 24 to February 10), and recovery times (February 11 to February 29).

In Fig. S5, the flowchart illustrates the extrapolation process: first, China Unicom’s users’ locations were extracted as a baseline for spatial aggregation; second, phone calls, messaging, and other types of communications between China Unicom and other operators’ users were weighted to estimate the number of users in the same district; third, district level user coverage ratios were summarized for each province; lastly, the real user coverage ratios (market share), which are available at the provincial level, were then used to train and obtain the optimized weighting at different districts in step two. We performed the F test to verify the fitting of this approach with real coverage ratios in four cities, as well as 31 provinces in China. The *P*-value of the F test was 0.999 and 0.584, respectively. Results show that there is no significant difference under confidence level of 0.05. Thus, the real and estimated coverage ratios are highly coincidental (Fig. S6).

In addition, our analysis indicates that there is a high agreement between the two numbers (i.e., number of users from the whole network and from the operator), and the ratio of the operator’s own sum ($m$) to the network estimates ($m^{*}$), $\theta\text{=}m/m^{*}$, varies from county to county. The correlation coefficients of *m* and $m^{*}$are all close to one at three admin levels: *r*=0.9999 for the county level, and *r*=1.0 for both the district level and the provincial level (Fig. S7).

**Data representativeness**

(1) Comparison of mobile phone estimates with population statistics.

The advantages of mobile phone data include extensive coverage and high representativeness. For China, the number of mobile phone subscriptions reached 1.6 billion by November 2019, with individuals owning more than one mobile phone number on average[[1]](#footnote-1). A total of 847 million Chinese people used mobile phones to surf the internet, accounting for 99.1% of the total netizens.

The penetration rate of mobile phone usage among the population aged 15–65 years is almost 100%. Estimation to the general population can then be made by extrapolating from the number of users in the whole network against the dependency ratio – the ratio of the population aged 0–14 and 65+ to those in the labor force (ages 15–64). According to the National Bureau of Statistics[[2]](#footnote-2), China has a youth dependency ratio of 23.7% and an old-age dependency ratio of 16.8%.

We used data provided by China Unicom to evaluate the consistency between the population estimated from mobile phone data with the above adjustments, and the official statistics on the residential population for central prefectures at the district level (Fig. S8). The estimated number of residents agrees well with the official statistics (*R*^2^=0.98).

(2) Comparison of mobile phone estimates with Baidu LBS data.

To investigate the difference between mobile phone data estimates and the flow generated by one of the most widely used open source, Baidu LBS (location-based services)[[3]](#footnote-3), we compared the top 10% of destination cities from both datasets, as presented in Fig. S9. Generally, the cities estimated with heavy amount of flows from Wuhan by Baidu LBS, were also top destination cities estimated by the mobile phone data. There is a high consistency for the first 20 to 30 cities outside Wuhan, and the overall Pearson correlation is *r* = 0.96 (*p*< 0.001).

Data processing and aggregation followed the laws of “Provisions on Protecting the Personal Information of Telecommunications and Internet Users (Mainland China)”[[4]](#footnote-4), and with reference to the GSMA (Global System for Mobile Communications Association) guidelines on the protection of privacy in the use of mobile phone data for responding to the Ebola outbreak[[5]](#footnote-5). The authors only had access to exported and aggregated data at the city level. All mobility data were provided by the operator for emergency responses and were anonymized and aggregated. No personally identifying information was processed in the analysis of this study.

**Mobility models**

1. Gravity model.

The gravity model was proposed to calculate mobility flows inspired by Newton’s law of universal gravitation, which emphasizes the impact of the distance between two places and the population on human migration. Likewise, the gravity model assumes that the population flow $T_{ij}$ between locations *i* and *j* is proportional to the population of *i* and *j* and inversely proportional to the distance between the two locations.

$$T_{ij}=\frac{m_{i}^{\alpha}n_{j}^{\beta}}{f\left(r_{ij}\right)},$$

where$m_{i}$and$n_{j}$represent the population of locations *m* and *n*, $\alpha$and$\beta$ are adjustable exponents, and the distance decay function $f\left(r_{ij}\right)$ is commonly modelled with an inverse power law or a negative exponential law form. We examined both forms as the distance-decay function with our mobility data, and the results support power-law distance decay. Thus, $f\left(r_{ij}\right)$can be expressed as:

$$f\left(r_{ij}\right)=r_{ij}^{\text{-}b},$$

the importance of distance in commuting choices is adjusted through parameter *b*, which is selected to maximize the fit between the flows estimated by the gravity model and the observed flows[[6]](#footnote-6).

1. Radiation model.

The radiation model was proposed based on the divergence and absorption process of material movement in solid physics. The radiation model assumes that an individual chooses a destination based on two steps. First, the individual evaluates the benefit of the location’s opportunities, and assigns a fitness parameter *z,* chosen from some distribution *p*(*z*). The individual then ranks all opportunities according to their distances from the origin location and chooses the closest one with a fitness higher than the individual’s fitness threshold, another random value extracted from *p*(*z*). Thus, the radiation model obtains the average number of travelers from position *i* to position *j*, expressed in the following form:

$$T_{ij}=\frac{m_{i}n_{j}}{\left(m_{i}+s_{ij}\right)\left(m_{i}+n_{j}+s_{ij}\right)},$$

where $T_{ij}$ is the total number of commuters from county *i*, $m_{i}$and$n_{j}$are the population in county *i* and *j*, respectively, and $s_{ij}$is the total population in the circle centered at *i* and touching *j* excluding the source and the destination population.

We use the RMSE to quantify the goodness of fit for the mobility models. Let $p(x)$and $p^{*}(x)$be the distribution of model outputs and real data, respectively, the RMSE is calculated as:

$$RMSE=\sqrt{\frac{1}{n}\sum_{x} (p(x)-p^{*}(x))^{2}}$$

**Louvain algorithm**

The Louvain algorithm is based on multi-level modularity optimization to extract the community structure of large networks. The algorithm is divided into two phases that are repeated iteratively. In the first phase, a partition of the given network is computed by modularity optimization. When the modularity reaches a local maximum, which means that no individual movement can improve the modularity, the first phase stop. In the second phase, the same community is folded to form a new weighted network. Once the second phase is completed, the first phase of the algorithm can then be reapplied to the resulting weighted network and iterated. The optimization objective of the algorithm is to maximize the modularity, and the definition of modularity is as follows:

$$Q=\frac{1}{2m}\sum_{i,j} \left[A_{ij}-\frac{k_{i}k_{j}}{2m}\right]\delta\left(c_{i},c_{j}\right),$$

where $A_{ij}$ represents the weight of the edge between *i* and *j*, $k_{i}=\sum_{j} A_{ij}$is the sum of the weights of edges attached to vector *i*,$c_{i}$is the community to which vector *i* is assigned, the$\delta\left(u,v\right)$is 1 if $u=v$ and 0 otherwise and $m=\frac{1}{2}\sum_{ij} A_{ij}$.


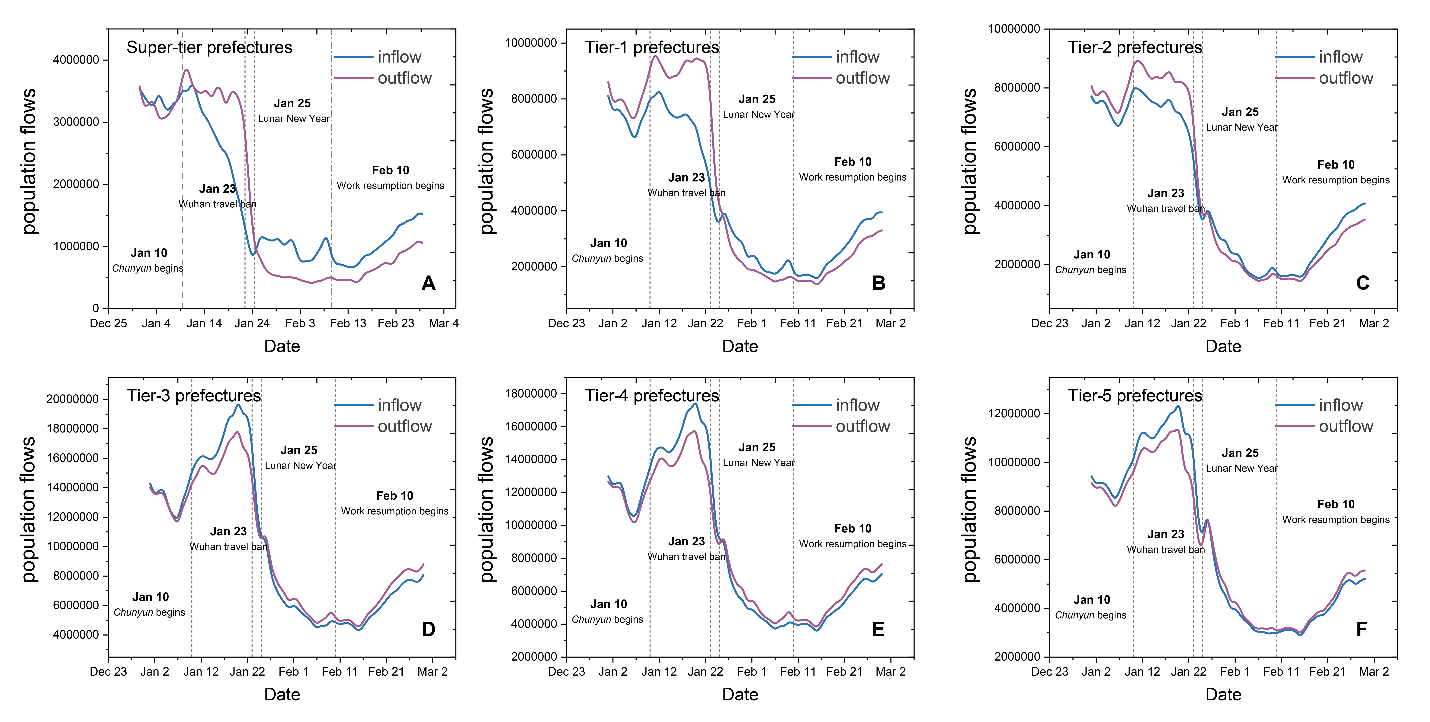


Fig. S1. Population flows across six prefecture tiers from January 1 to February 29, 2020. The inflow and outflow are shown for each prefecture tier. The variance of mobility around the population flows differed between prefectures, which were grouped into (A) Super-tier, (B) Tier-1, (C) Tier-2, (D) Tier-3, (E) Tier-4, and (F) Tier-5.


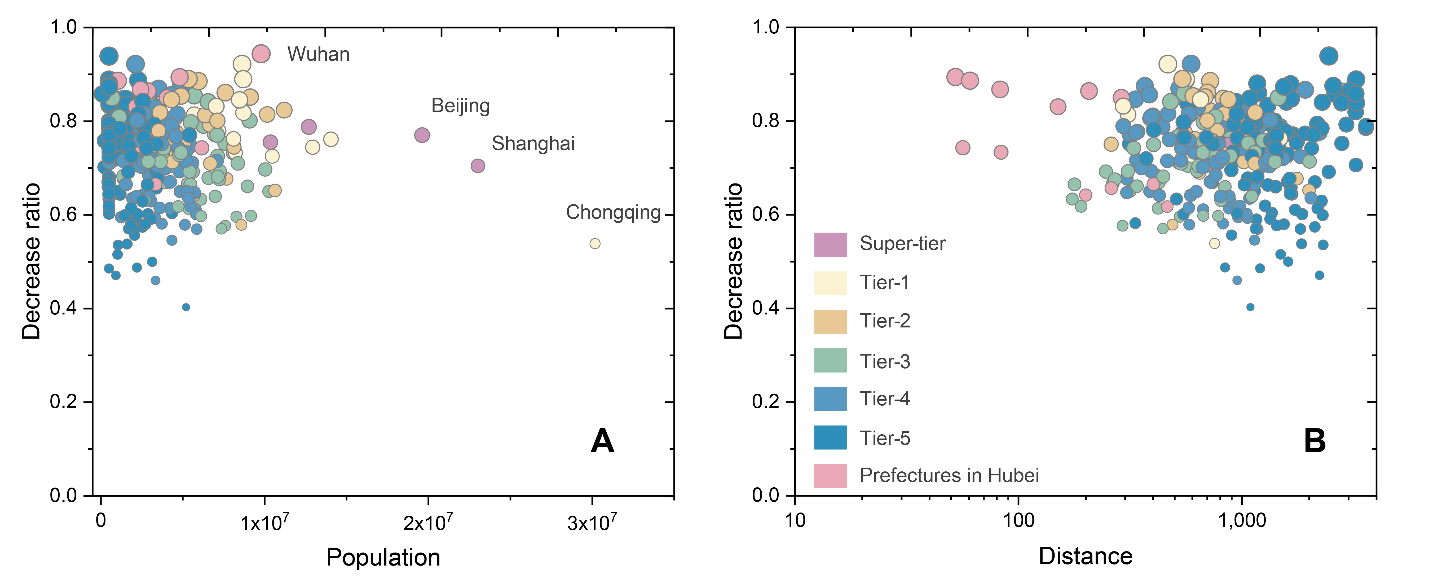


Fig. S2. Factors correlated with the decrease ratio. (A) The association with prefecture population. (B) The association with distance from Wuhan. The size of the node is proportional to the decrease ratio.


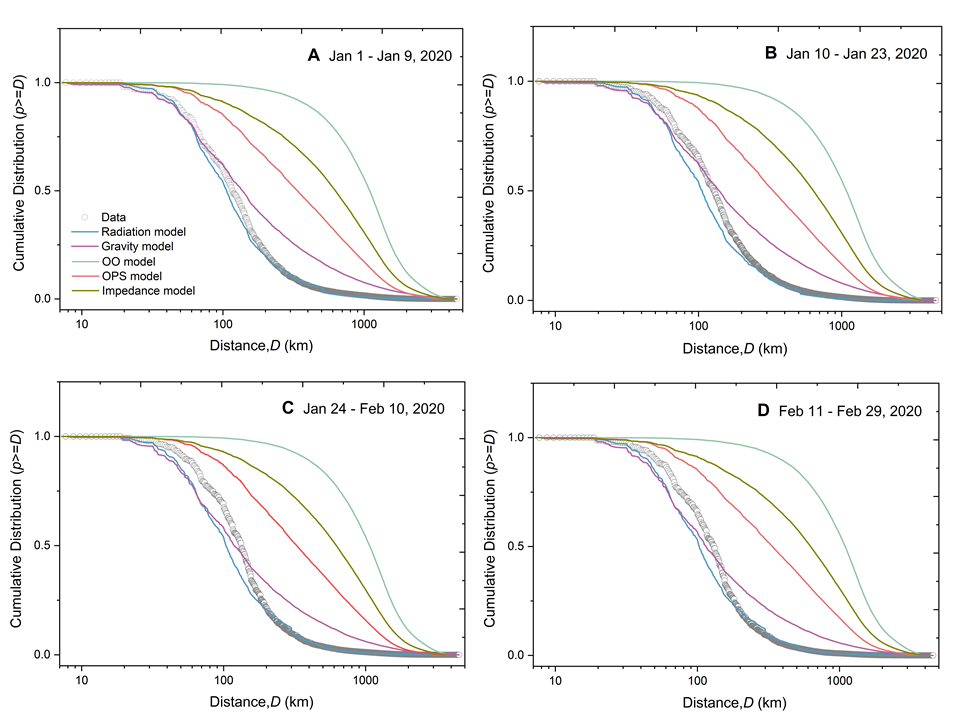


Fig. S3. Travel distance distributions produced by the Gravity model, the Radiation model, the OPS model[[7]](#footnote-7), the OO model[[8]](#footnote-8), and the impedance model[[9]](#footnote-9) compared with real data. (A) January 1 to January 9, 2020. (B) January 10 to January 23, 2020. (C) January 24 to February 10, 2020. (D) February 11 to February 29, 2020. *p* is defined as the probability of travel between locations at a certain distance. The OPS model assumes that an individual will select destination locations that present higher opportunity benefits than the location opportunities of the origin and the intervening opportunities between the origin and destination. In the OO model, the individual chooses the location whose benefit is higher than the benefit of the origin. These two models, as well as the classical radiation model (the individual only chooses the closest potential destination), characterize three types of the individual’s destination selection behavior. In addition, we tested the impedance model, which has been proposed to model the mobility of the population during the cholera outbreak in Haiti. However, as the mobility activities in China experienced extreme fluctuations during *chunyun* and the lockdown, the result indicates that these methods all failed to illustrate the mobility patterns supporting that *chunyun*, the pandemic, and lockdown are intertwined.


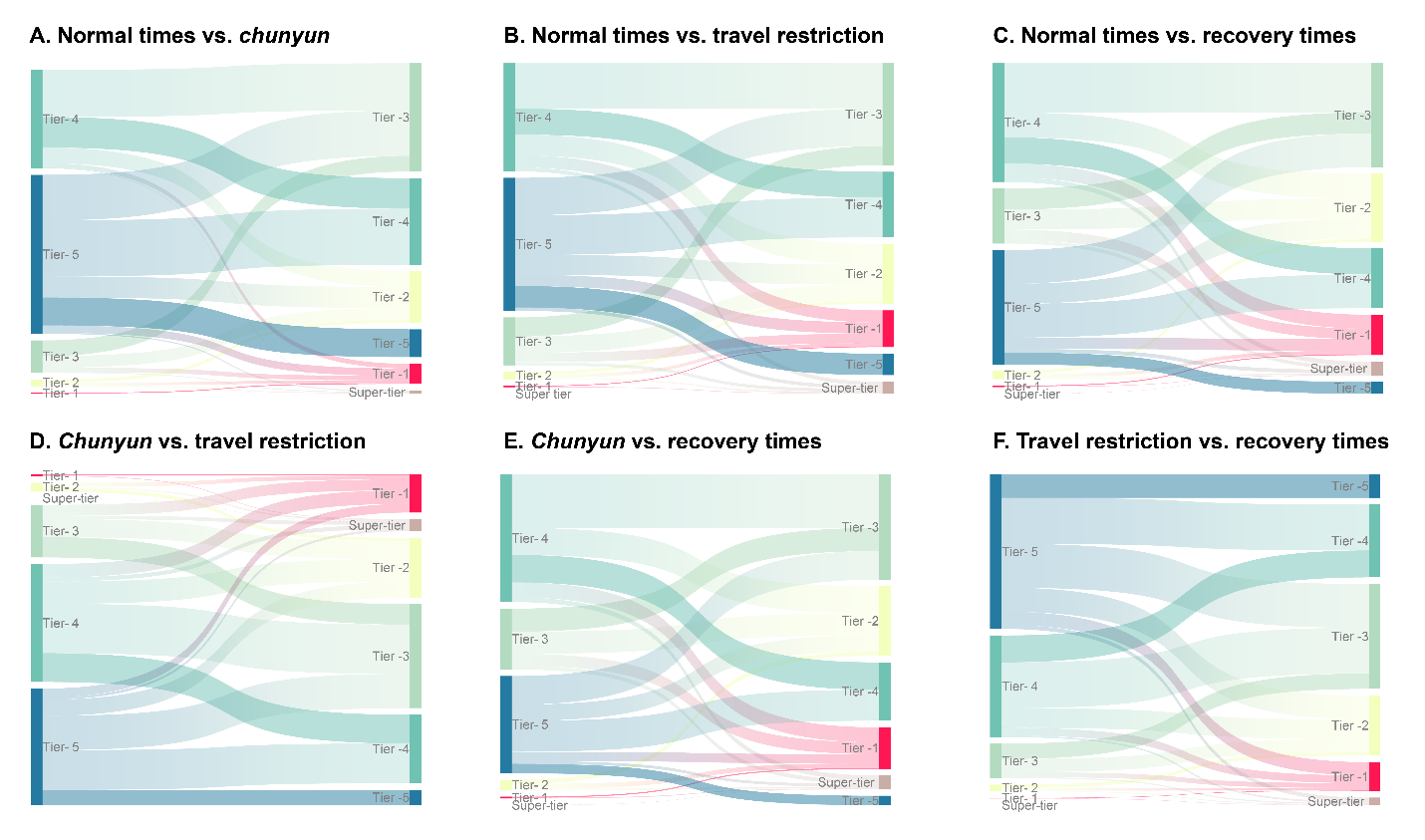


Fig. S4. Alluvial diagrams for mapping changes in backflow. The six prefecture tiers are ordered and shown as blocks. Normal times: January 1 to January 9; *chunyun*: January 10 to January 23; Travel restrictions: January 24 to February 10; Recovery times: February 11 to February 29. The height of a streamline between two stages is proportional to the number of backflow migration movements that occurred every two phases.


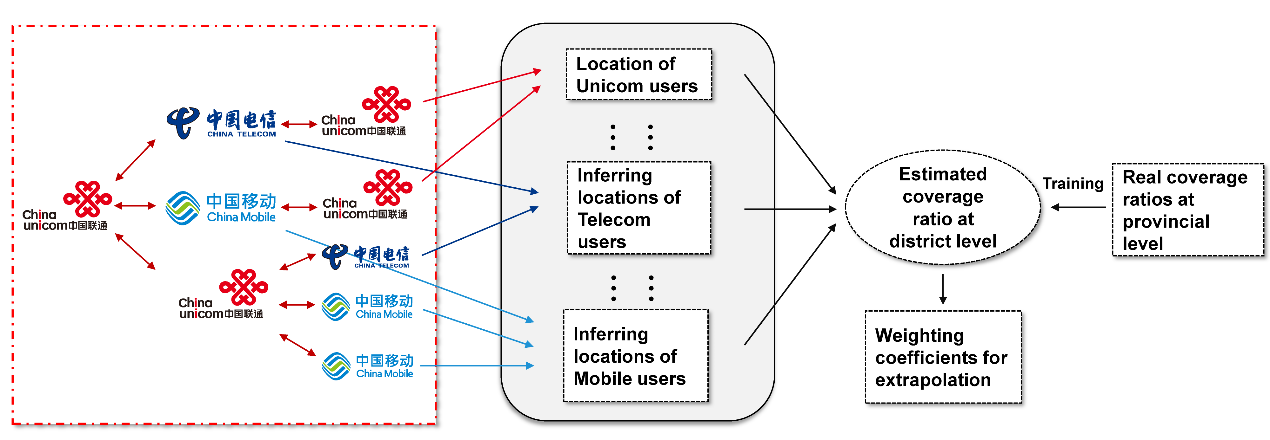


Fig. S5. Schematic chart for the workflow in data extrapolation.


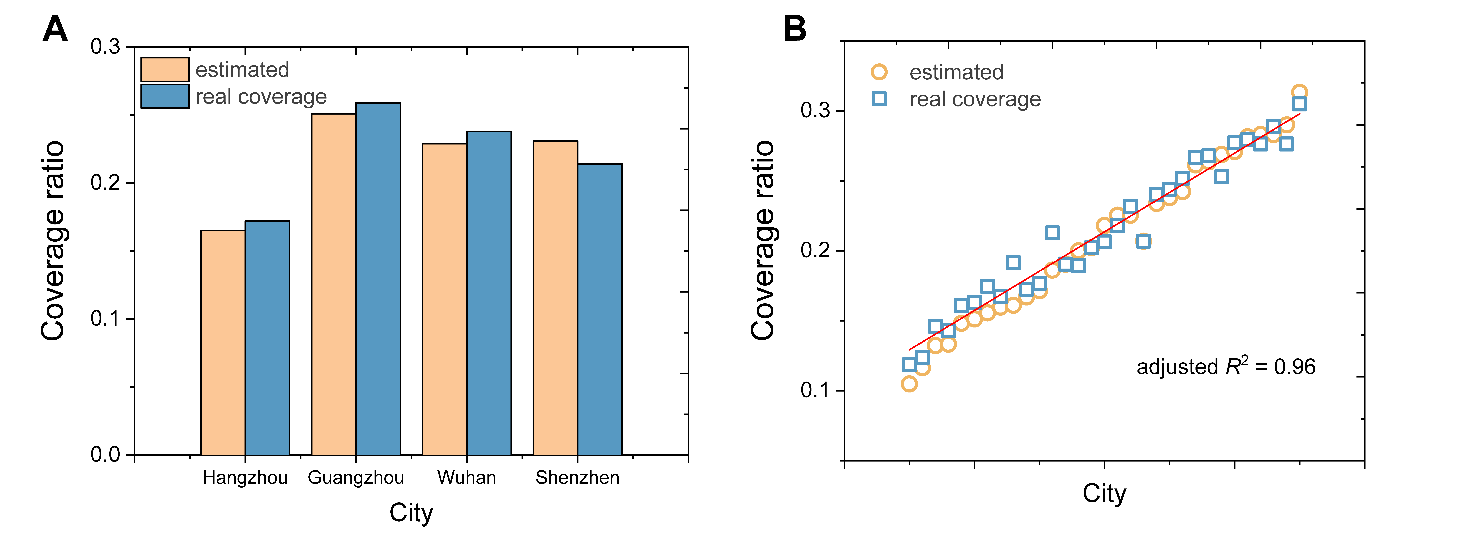


Fig. S6. Validation of data extrapolation. (A) Comparison of real and estimated user coverage ratios in four selected cities: Hangzhou, Wuhan, Guangzhou and Shenzhen. (B) Comparison of real and estimated user coverage ratios in 31 provinces. Data provided by China Unicom.


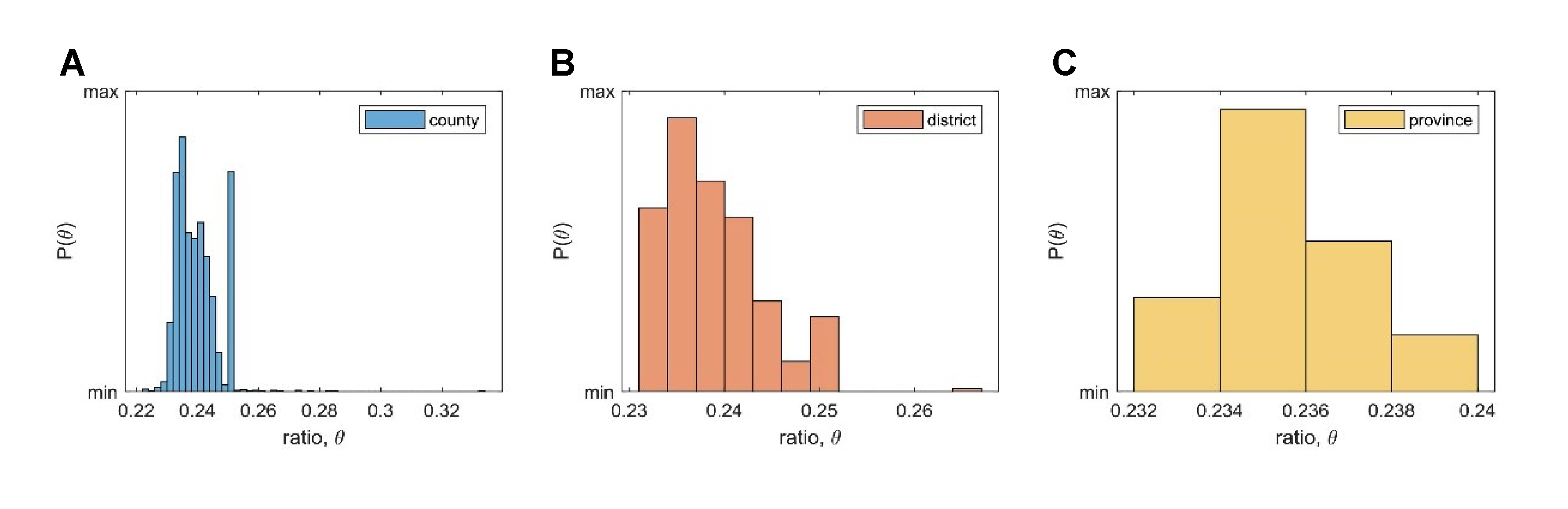


Fig. S7. Ratio of the estimated number of users from the whole network, to the number of users from the operator. (A) County level; (B) District level; (C) Provincial level.


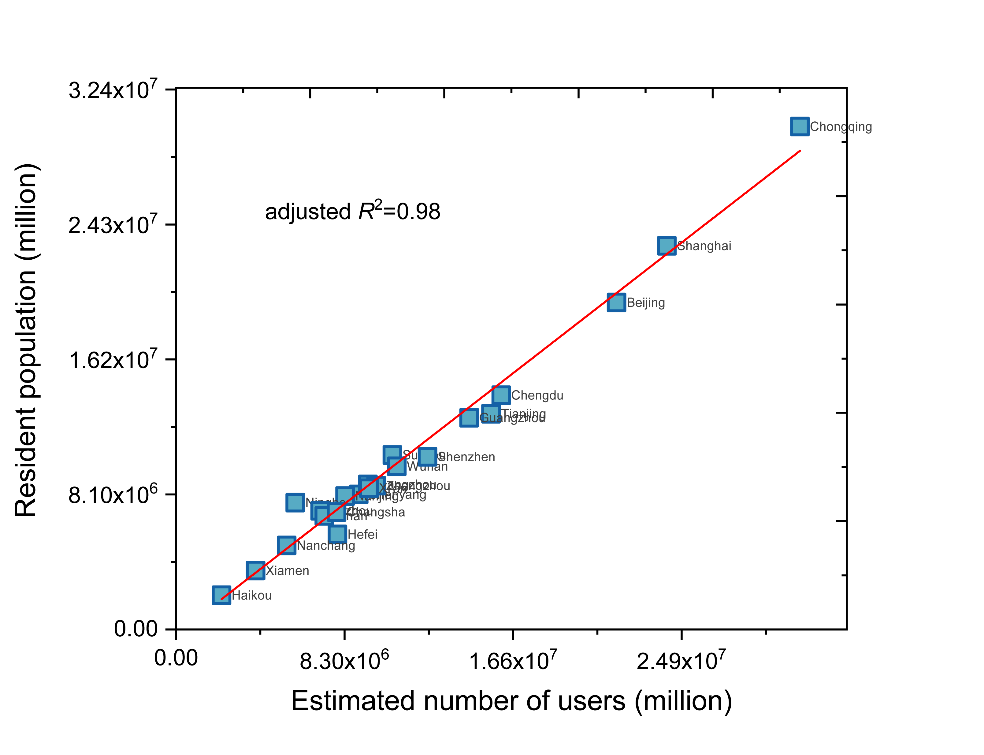


Fig. S8. Consistency of mobile phone users’ estimates with the population data for central prefectures at the district level. Data provided by China Unicom.


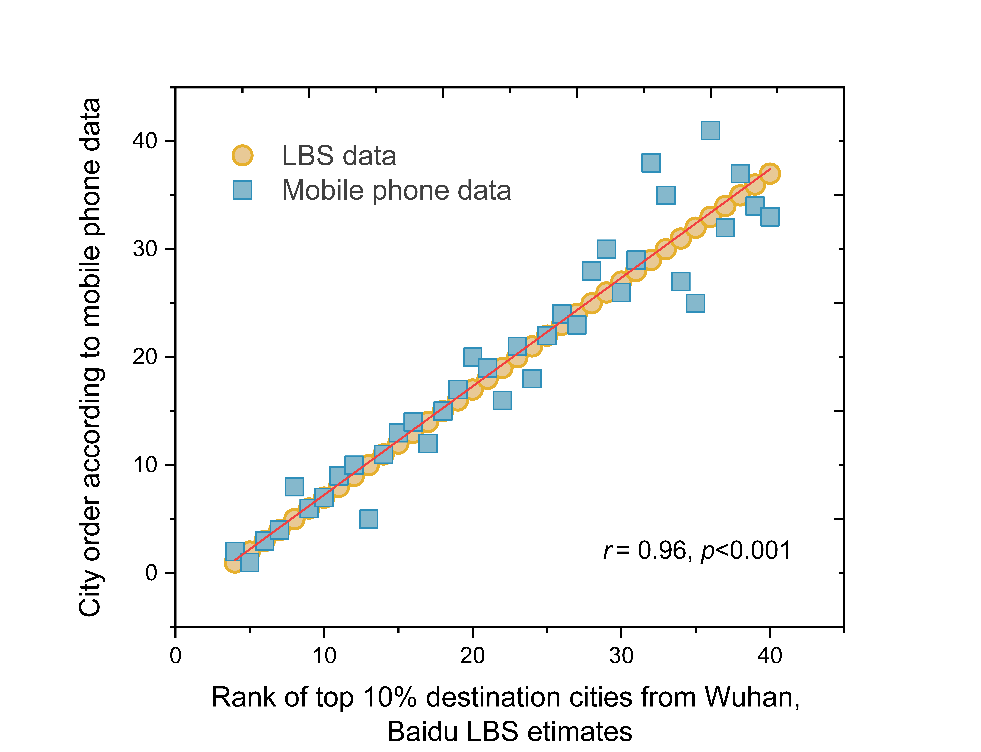


Fig. S9. Comparison of the order of the top 10% destination cities from Wuhan on January 22, 2020 (the day before the lockdown), based on population flows estimated from China Unicom and Baidu data.

Table S1. Summary of travel distances for different periods (distance: km).

| Percentile（%） | Jan1–Jan9 | Jan10–Jan23 | Jan24–Feb9 | Feb11–Feb29 |
| --- | --- | --- | --- | --- |
| 10 | 47 | 50 | 53 | 51 |
| 20 | 63 | 67 | 70 | 68 |
| 30 | 77 | 87 | 96 | 89 |
| 40 | 100 | 107 | 114 | 109 |
| 50 | 116 | 128 | 133 | 131 |
| 60 | 140 | 148 | 149 | 148 |
| 70 | 163 | 176 | 168 | 165 |
| 80 | 208 | 223 | 206 | 202 |
| 90 | 306 | 338 | 296 | 293 |
| 99 | 1235 | 1213 | 1124 | 941 |

Table S2. Comparison of the root mean squared errors of the fit for the five mobility models.

| Mobility models | Jan1–Jan9 | Jan10–Jan23 | Jan24–Feb9 | Feb11–Feb29 |
| --- | --- | --- | --- | --- |
| Gravity model | 0.055 | 0.054 | 0.046 | 0.044 |
| Radiation model | 0.010 | 0.017 | 0.021 | 0.018 |
| OPS model | 0.178 | 0.172 | 0.178 | 0.176 |
| OO model | 0.464 | 0.456 | 0.462 | 0.460 |
| Impedance model | 0.289 | 0.285 | 0.296 | 0.292 |

Table S3. Statistics of the population flows produced by daily commuting. Large population flows usually exists in neighboring prefectures that are geographically connected, and are generated by daily commuting rather than backflowing. Here we list the top five population netflows with the prefectures pair interactions that existed across the four periods.

| Origin | Destination | Jan1–Jan9 | Jan10–Jan23 | Jan24–Feb9 | Feb11–Feb29 |
| --- | --- | --- | --- | --- | --- |
| Shanghai  (31º23´N, 121º47´E) | Suzhou  (Jiangsu)  (31º29´N, 120º61´E) | –423,938 | –462,571 | –378,532 | –282,194 |
| Beijing  (39º56´N, 116º20´E) | Langfang  (39º52´N, 116º71´E) | –846,641 | –1,070,641 | –231,455 | –286,504 |
| Guangzhou  (23º13´N, 113º28´E) | Foshan  (23º02´N, 113º12´E) | –406,696 | –742,273 | –85,157 | –107,832 |
| Suzhou  (Anhui)  (33º63´N, 116º98´E) | Xuzhou  (34º26´N, 117º18´E) | 252,609 | 440,174 | 795,335 | 937,067 |
| Liangshan  (27º88´N, 102º26´E) | Zhaotong  (27º33´N, 103º71´E) | –531,441 | –877,557 | –880,016 | –868,707 |

Table S4. Members of communities detected in normal times.

| Communities | Members | Central prefectures |
| --- | --- | --- |
| 1-Southwest  (Chengdu) | Chongqing, Chengdu, Guiyang, Kunming Luzhou, Zhaotong, Liangshan Yi Autonomous Prefecture, Yibin, Bijie, Guangan, Zunyi, Dazhou, Nanchong Neijiang, Mianyang, Ziyang, Deyang, Meishan Suining, Zigong, Liupanshui, Qujing, Leshan Bazhong, Tongren, Xiangxi Tujia and Miao Autonomous Prefecture, Guangyuan, Enshi Tujia and Miao Autonomous Prefecture, Qiannan Buyi and Miao Autonomous Prefecture, Qiandongnan Miao and Dong Autonomous Prefecture, Anshun, Qianxinan Buyi and Miao Autonomous Prefecture, Huaihua, Ya'an, Dali Bai Autonomous Prefecture, Panzhihua, Honghe Hani Yi Autonomous Prefecture, Chuxiong Yi Autonomous Prefecture, Lijiang, Hanzhong, Yuxi, Wenshan Zhuang and Miao Autonomous Prefecture, Lincang Aba Tibetan and Qiang Autonomous Prefecture, Pu'er, Dehong Dai Jingpo Autonomous Prefecture, Xishuangbanna Dai Autonomous Prefecture, Ganzi Tibetan Autonomous Prefecture, Diqing Tibetan Autonomous Prefecture, Nujiang Lisu Autonomous Prefecture, Changdu | Chengdu  Chongqing Guiyang |
| 2-Northeast | Shenyang, Changchun, Harbin, Suihua, Qiqihar, Anshan, Daqing, Tongliao, Siping, Tieling, Chaoyang, Jinzhou, Chifeng, Liaoyang, Songyuan, Fuxin, Panjin, Huludao, Xingan League, Jilin, Dalian, Heihe, Fushun, Baicheng, Yingkou, Liaoyuan, Hulunbeir, Tonghua, Benxi, Jiamusi, Dandong, Yichun, Mudanjiang, Yanbian Korean Autonomous Prefecture, Shuangyashan, Baishan, Qitaihe, Hegang, Jixi, Daxing'anling | Shenyang Changchun Harbin |
| 3-Central China | Zhengzhou, Xuzhou, Fuyang, Zhoukou, Suzhou, Shangqiu, Bozhou, Heze, Jining, Zhumadian, Linyi, Xinyang, Nanyang, Kaifeng, Xinxiang, Xuchang, Xiangyang, Zaozhuang, Huaibei, Luoyang, Pingdingshan, Luohe, Rizhao, Jiaozuo, Shiyan, Ankang, Shennongjia | Zhengzhou |
| 4-Jing-Jin-Ji | Beijing, Tianjin, Jinan, Shijiazhuang, Handan, Langfang, Liaocheng, Dezhou, Puyang, Cangzhou, Anyang, Xingtai, Yantai, Baoding, Binzhou, Hengshui, Qingdao, Zibo, Weifang, Taian, Tangshan, Weihai, Zhangjiakou, Chengde, Dongying, Qinhuangdao, Hebi, Xilingole League | Beijing Tianjin Shijiazhuang Jinan |
| 5-Yangtze River Delta | Shanghai, Nanjing, Hefei, Hangzhou, Suzhou, Anqing, Wuxi, Chuzhou, Lu'an, Changzhou, Jiaxing, Taizhou, Yancheng, Suqian, Ma’anshan, Lianyungang, Nantong, Chizhou, Yangzhou, Huaian, Zhenjiang, Bengbu, Wuhu, Xuancheng, Huainan, Shaoxing, Ningbo, Wenzhou, Huzhou, Jinhua, Tongling, Quzhou, Taizhou, Huangshan, Lishui, Zhoushan | Shanghai Nanjing Hefei Hangzhou |
| 6-Pearl River Delta | Guangzhou, Shenzhen, Dongguan, Foshan, Zhongshan, Huizhou, Jieyang, Shantou, Zhuhai, Zhaoqing, Jiangmen, Qingyuan, Chaozhou, Shaoguan, Yunfu, Shanwei, Meizhou, Heyuan, Heyuan, Yangjiang | Guangzhou Shenzhen Dongguan Foshan |
| 7-South (Haikou) | Haikou, Sanya, Chengmai, Wenchang, Danzhou, Ding'an, Qionghai, Wanning, Lingshui Li Autonomous County, Lingao, Ledong Li Autonomous County, Dongfang, Tunchang, Changjiang Li Autonomous County, Baisha Li Autonomous County, Baoting Li and Miao Autonomous County, Qiongzhong Li and Miao Autonomous County, Wuzhishan, Sansha | Haikou Sanya |
| 8-Northwest  (Urumqi) | Urumqi, Kashgar, Changji Hui Autonomous Prefecture, Kizilsu Kirgiz Autonomous Prefecture, Ili Kazakh Autonomous Prefecture, Bayingolin Mongolian Autonomous Prefecture, Tacheng, Shihezi, Tumushuke, Tiemenguan, Aksu, Aletai, Wujiaqu, Beitun, Karamay, Bortala Mongolian Autonomous Prefecture, Hotan, Turpan, Hami, Alaer, KekeDala, Shuanghe, Kunyu | Urumqi |
| 9-South Central  (Wuhan) | Changsha, Xiamen, Nanchang, Fuzhou, Yueyang, Jingzhou, Huanggang, Jiujiang, Shangrao, Hengyang, Yichun, Quanzhou, Changde, Zhangzhou, Shaoyang, Yongzhou, Zhuzhou, Ganzhou, Xiangtan, Chenzhou, Yiyang, Xiaogan, Loudi, Pingxiang, Ningde, Ji'an, Xianning, Huangshi, Putian, Ezhou, Jingmen, Yichang, Longyan Sanming, Jingdezhen, Xiantao, Tianmen, Nanping, Suizhou, Hezhou, Yingtan, Xinyu, Zhangjiajie, Qianjiang | Wuhan Changsha Xiamen |
| 10-Northwest  (Xi’an) | Xi'an, Taiyuan, Lanzhou, Xining, Yinchuan, Lhasa, Hohhot, Xianyang, Baotou, Yulin, Yuncheng, Weinan, Luliang, Ordos, Sanmenxia, Xinzhou, Haidong, Jinzhong, Pingliang, Linfen, Ulanqab, Qingyang, Wuzhong, Dingxi, Baoji, Linxia Hui Autonomous Prefecture, Zhongwei, Tianshui, Longnan, Guyuan, Baiyin Yanan, Shuozhou, Changzhi, Wuhai, Shizuishan, Shangluo, Gannan Tibetan Autonomous Prefecture, Wuwei, Jincheng, Tongchuan, Yangquan, Bayan Nur, Jiuquan, Alxa League, Tibetan Autonomous Prefecture of Huangnan, Jiayuguan, Jinchang, Zhangye, Shannan, Hainan Tibetan Autonomous Prefecture, Haibei Tibetan Autonomous Prefecture, Shigatse, Haixi Mongolian and Tibetan Autonomous Prefecture, Naqu, Linzhi, Yushu Tibetan Autonomous Prefecture, Ali, Golog Tibetan Autonomous Prefecture | Xi’an Taiyuan Lanzhou |
| 11-South （Nanning） | Nanning, Maoming, Zhanjiang, Yulin, Liuzhou, Guigang, Baise, Guilin, Laibin, Hechi, Qinzhou, Beihai, Wuzhou, Chongzuo, Fangchenggang | Nanning |

Table S5. Members of communities detected in *chunyun* migration.

| Communities | Members | Central prefectures |
| --- | --- | --- |
| 1-Southwest  (Chengdu) | Chongqing, Chengdu, Guiyang, Kunming Luzhou, Zhaotong, Liangshan Yi Autonomous Prefecture, Yibin, Bijie, Guangan, Zunyi, Dazhou, Nanchong, Neijiang, Mianyang, Ziyang, Deyang, Meishan, Suining, Zigong, Liupanshui, Qujing, Leshan, Bazhong, Tongren, Xiangxi Tujia and Miao Autonomous Prefecture, Guangyuan, Enshi Tujia and Miao Autonomous Prefecture, Qiannan Buyi and Miao Autonomous Prefecture, Qiandongnan Miao and Dong Autonomous Prefecture, Anshun, Qianxinan Buyi and Miao Autonomous Prefecture, Huaihua, Ya'an, Dali Bai Autonomous Prefecture, Panzhihua, Honghe Hani Yi Autonomous Prefecture, Chuxiong Yi Autonomous Prefecture, Lijiang, Hanzhong, Yuxi, Wenshan Zhuang and Miao Autonomous Prefecture, Lincang, Baoshan, Aba Tibetan and Qiang Autonomous Prefecture, Pu'er, Dehong Dai Jingpo Autonomous Prefecture, Xishuangbanna Dai Autonomous Prefecture, Ganzi Tibetan Autonomous Prefecture, Diqing Tibetan Autonomous Prefecture, Nujiang Lisu Autonomous Prefecture, Changdu, Zhangjiajie, Lhasa, Shannan, Shigatse, Naqu, Linzhi, Ali, Baise | Chengdu  Chongqing Guiyang |
| 2-Northeast | Shenyang, Changchun, Harbin, Suihua, Qiqihar, Anshan, Daqing, Tongliao, Siping, Tieling, Chaoyang, Jinzhou, Chifeng, Liaoyang, Songyuan, Fuxin, Panjin, Huludao, Xingan League, Jilin, Dalian, Heihe, Fushun, Baicheng, Yingkou, Liaoyuan, Hulunbeir, Tonghua, Benxi, Jiamusi, Dandong, Yichun, Mudanjiang, Yanbian Korean Autonomous Prefecture, Shuangyashan, Baishan, Qitaihe, Hegang, Jixi, Daxing'anling | Shenyang Changchun Harbin |
| 3-Central China | Zhengzhou, Xuzhou, Fuyang, Zhoukou, Suzhou, Shangqiu, Bozhou, Heze, Jining, Zhumadian, Linyi, Xinyang, Kaifeng, Xinxiang, Xuchang, Zaozhuang, Huaibei, Luoyang, Pingdingshan, Luohe, Rizhao, Jiaozuo, Hefei, Anqing, Lu'an, Suqian, Lianyungang, Chizhou, Huaian, Huainan, Tongling | Zhengzhou Hefei |
| 4-Jing-Jin-Ji | Beijing, Tianjin, Jinan, Shijiazhuang, Handan, Langfang, Liaocheng, Dezhou, Puyang, Cangzhou, Anyang, Xingtai, Yantai, Baoding, Binzhou, Hengshui, Qingdao, Zibo, Weifang, Taian, Tangshan, Weihai, Zhangjiakou, Chengde, Dongying, Qinhuangdao, Hebi, Xilingole League, Shuozhou, Datong | Beijing Tianjin Shijiazhuang Jinan |
| 5-Yangtze River Delta | Shanghai, Nanjing, Hangzhou, Suzhou, Wuxi, Chuzhou, Changzhou, Jiaxing, Taizhou, Yancheng, Ma’anshan, Nantong, Yangzhou, Zhenjiang, Bengbu, Wuhu, Xuancheng, Shaoxing, Ningbo, Huzhou, Jinhua, Taizhou, Huangshan, Zhoushan | Shanghai Nanjing Hangzhou |
| 6-Pearl River Delta | Guangzhou, Shenzhen, Dongguan, Foshan, Zhongshan, Huizhou, Jieyang, Shantou, Zhuhai, Zhaoqing, Jiangmen, Qingyuan, Chaozhou, Yunfu, Shanwei, Meizhou, Heyuan, Heyuan | Guangzhou Shenzhen Dongguan Foshan |
| 7-South (Haikou) | Haikou, Sanya, Chengmai, Wenchang, Danzhou, Ding'an, Qionghai, Wanning, Lingshui Li Autonomous County, Lingao, Ledong Li Autonomous County, Dongfang, Tunchang, Changjiang Li Autonomous County, Baisha Li Autonomous County, Baoting Li and Miao Autonomous County, Qiongzhong Li and Miao Autonomous County, Wuzhishan, Sansha, Yangjiang, Nanning, Maoming, Zhanjiang, Yulin, Liuzhou, Guigang, Laibin, Hechi, Qinzhou, Beihai, Wuzhou, Chongzuo, Fangchenggang | Haikou Nanning |
| 8-Northwest  (Urumqi) | Urumqi, Kashgar, Changji Hui Autonomous Prefecture, Kizilsu Kirgiz Autonomous Prefecture, Ili Kazakh Autonomous Prefecture, Bayingolin Mongolian Autonomous Prefecture, Tacheng, Shihezi, Tumushuke, Tiemenguan, Aksu, Aletai, Wujiaqu, Beitun, Karamay, Bortala Mongolian Autonomous Prefecture, Hotan, Turpan, Hami, Alaer, KekeDala, Shuanghe, Kunyu | Urumqi |
| 9-South Central  (Wuhan) | Wuhan, Changsha, Nanchang, Yueyang, Jingzhou, Huanggang, Jiujiang, Shangrao, Hengyang, Yichun, Changde, Shaoyang, Yongzhou, Zhuzhou, Ganzhou, Xiangtan, Chenzhou, Yiyang, Xiaogan, Loudi, Pingxiang, Ji'an, Xianning, Huangshi, Ezhou, Jingmen, Yichang, Jingdezhen, Xiantao, Tianmen, Suizhou, Hezhou, Yingtan, Xinyu, Qianjiang, Shennongjia, Nanyang, Shiyan, Ankang, Xiangyang, Quzhou, Shaoguan, Guilin | Wuhan Changsha |
| 10-Northwest  (Xi’an) | Xi'an, Taiyuan, Lanzhou, Xining, Yinchuan, Hohhot, Xianyang, Baotou, Yulin, Yuncheng, Weinan, Luliang, Ordos, Sanmenxia, Xinzhou, Haidong, Jinzhong, Pingliang, Linfen, Ulanqab, Qingyang, Wuzhong, Dingxi, Baoji, Linxia Hui Autonomous Prefecture, Zhongwei, Tianshui, Longnan, Guyuan, Baiyin Yanan, Changzhi, Wuhai, Shizuishan, Shangluo, Gannan Tibetan Autonomous Prefecture, Wuwei, Jincheng, Tongchuan, Yangquan, Bayan Nur, Jiuquan, Alxa League, Tibetan Autonomous Prefecture of Huangnan, Jiayuguan, Jinchang, Zhangye, Hainan Tibetan Autonomous Prefecture, Haibei Tibetan Autonomous Prefecture, Haixi Mongolian and Tibetan Autonomous Prefecture, Yushu Tibetan Autonomous Prefecture, Golog Tibetan Autonomous Prefecture | Xi’an Taiyuan Lanzhou |
| 11- South Central（Xiamen） | Xiamen, Fuzhou, Quanzhou, Zhangzhou, Ningde, Putian, Longyan, Sanming, Nanping, Wenzhou, Lishui | Xiamen |

Table S6. Members of communities detected in travel restrictions.

| Communities | Members | Central prefectures |
| --- | --- | --- |
| 1-Southwest  (Chengdu) | Chongqing, Chengdu, Guiyang, Kunming Luzhou, Zhaotong, Liangshan Yi Autonomous Prefecture, Yibin, Bijie, Guangan, Zunyi, Dazhou, Nanchong, Neijiang, Mianyang, Ziyang, Deyang, Meishan, Suining, Zigong, Liupanshui, Qujing, Leshan, Bazhong, Tongren, Xiangxi Tujia and Miao Autonomous Prefecture, Guangyuan, Enshi Tujia and Miao Autonomous Prefecture, Qiannan Buyi and Miao Autonomous Prefecture, Qiandongnan Miao and Dong Autonomous Prefecture, Anshun, Qianxinan Buyi and Miao Autonomous Prefecture, Huaihua, Ya'an, Dali Bai Autonomous Prefecture, Panzhihua, Honghe Hani Yi Autonomous Prefecture, Chuxiong Yi Autonomous Prefecture, Lijiang, Baoshan, Hanzhong, Yuxi, Wenshan Zhuang and Miao Autonomous Prefecture, Baoshan, Lincang, Aba Tibetan and Qiang Autonomous Prefecture, Pu'er, Dehong Dai Jingpo Autonomous Prefecture, Xishuangbanna Dai Autonomous Prefecture, Ganzi Tibetan Autonomous Prefecture, Diqing Tibetan Autonomous Prefecture, Nujiang Lisu Autonomous Prefecture, Zhangjiajie, Baise, Liuzhou, Laibin, Hechi | Chengdu  Chongqing Guiyang |
| 2-Northeast | Shenyang, Changchun, Harbin, Suihua, Qiqihar, Anshan, Daqing, Tongliao, Siping, Tieling, Jinzhou, Liaoyang, Songyuan, Fuxin, Panjin, Huludao, Xingan League, Jilin, Dalian, Heihe, Fushun, Baicheng, Yingkou, Liaoyuan, Hulunbeir, Tonghua, Benxi, Jiamusi, Dandong, Yichun, Mudanjiang, Yanbian Korean Autonomous Prefecture, Shuangyashan, Baishan, Qitaihe, Hegang, Jixi, Daxing'anling | Shenyang Changchun Harbin |
| 3-Central China | Zhengzhou, Xuzhou, Fuyang, Zhoukou, Suzhou, Shangqiu, Bozhou, Heze, Jining, Zhumadian, Linyi, Xinyang, Kaifeng, Xinxiang, Xuchang, Zaozhuang, Huaibei, Luoyang, Pingdingshan, Luohe, Rizhao, Jiaozuo, Hefei, Lu'an, Suqian, Lianyungang, Huainan, Bengbu | Zhengzhou Hefei |
| 4-Jing-Jin-Ji | Beijing, Tianjin, Jinan, Shijiazhuang, Handan, Langfang, Liaocheng, Dezhou, Puyang, Cangzhou, Anyang, Xingtai, Yantai, Baoding, Binzhou, Hengshui, Qingdao, Zibo, Weifang, Taian, Tangshan, Weihai, Zhangjiakou, Chengde, Dongying, Qinhuangdao, Hebi, Xilingole League, Shuozhou, Datong, Chaoyang, Chifeng, Hohhot, Baotou, Ulanqab, Bayan Nur | Beijing Tianjin Shijiazhuang Jinan |
| 5-Yangtze River Delta | Shanghai, Nanjing, Hangzhou, Suzhou, Wuxi, Chuzhou, Changzhou, Jiaxing, Taizhou, Yancheng, Ma’anshan, Nantong, Yangzhou, Zhenjiang, Wuhu, Xuancheng, Shaoxing, Ningbo, Huzhou, Jinhua, Taizhou, Huangshan, Zhoushan, Huaian, Xiamen, Fuzhou, Quanzhou, Zhangzhou, Ningde, Putian, Longyan, Sanming, Nanping, Wenzhou, Lishui | Shanghai Nanjing Hangzhou Xiamen |
| 6-Pearl River Delta | Guangzhou, Shenzhen, Dongguan, Foshan, Zhongshan, Huizhou, Jieyang, Shantou, Zhuhai, Zhaoqing, Jiangmen, Qingyuan, Chaozhou, Yunfu, Shanwei, Meizhou, Heyuan, Heyuan, Nanning, Maoming, Zhanjiang, Yulin, Guigang, Qinzhou, Beihai, Wuzhou, Chongzuo, Fangchenggang, Yangjiang, Changsha, Yueyang, Jingzhou, Hengyang Changde, Shaoyang, Yongzhou, Zhuzhou, Xiangtan, Chenzhou, Yiyang, Loudi, Pingxiang, Guilin, Xianning, Shaoguan, Jingmen, Yichang, Xiantao Tianmen, Hezhou, Qianjiang | Guangzhou Shenzhen Dongguan Foshan Changsha |
| 7-South (Haikou) | Haikou, Sanya, Chengmai, Wenchang, Danzhou, Ding'an, Qionghai, Wanning, Lingshui Li Autonomous County, Lingao, Ledong Li Autonomous County, Dongfang, Tunchang, Changjiang Li Autonomous County, Baisha Li Autonomous County, Baoting Li and Miao Autonomous County, Qiongzhong Li and Miao Autonomous County, Wuzhishan, Sansha | Haikou |
| 8-Northwest  (Urumqi) | Urumqi, Kashgar, Changji Hui Autonomous Prefecture, Kizilsu Kirgiz Autonomous Prefecture, Ili Kazakh Autonomous Prefecture, Bayingolin Mongolian Autonomous Prefecture, Tacheng, Shihezi, Tumushuke, Tiemenguan, Aksu, Aletai, Wujiaqu, Beitun, Karamay, Bortala Mongolian Autonomous Prefecture, Hotan, Turpan, Hami, Alaer, KekeDala, Shuanghe, Kunyu | Urumqi |
| 9-South Central  (Wuhan) | Wuhan, Nanchang, Huanggang, Jiujiang, Shangrao, Yichun, Ganzhou, Xiaogan, Ji'an, Huangshi, Ezhou, Jingdezhen, Tianmen, Yingtan, Xinyu, Qianjiang, Quzhou, Anqing, Chizhou, Tongling | Wuhan |
| 10-Northwest  (Xi’an) | Xi'an, Taiyuan, Lanzhou, Xining, Yinchuan, Xianyang, Yulin, Yuncheng, Weinan, Luliang, Ordos, Sanmenxia, Xinzhou, Haidong, Jinzhong, Pingliang, Linfen, Qingyang, Wuzhong, Dingxi, Baoji, Linxia Hui Autonomous Prefecture, Zhongwei, Tianshui, Longnan, Guyuan, Baiyin Yanan, Changzhi, Wuhai, Shizuishan, Shangluo, Gannan Tibetan Autonomous Prefecture, Wuwei, Jincheng, Tongchuan, Yangquan, Jiuquan, Alxa League, Tibetan Autonomous Prefecture of Huangnan, Jiayuguan, Jinchang, Zhangye, Hainan Tibetan Autonomous Prefecture, Haibei Tibetan Autonomous Prefecture, Haixi Mongolian and Tibetan Autonomous Prefecture, Yushu Tibetan Autonomous Prefecture, Golog Tibetan Autonomous Prefecture, Shennongjia, Nanyang, Shiyan, Ankang, Xiangyang, Suizhou | Xi’an Taiyuan Lanzhou |
| 11-Southwest（Lhasa） | Lhasa, Shannan, Shigatse, Naqu, Linzhi, Ali, Changdu | Lhasa |

Table S7. Members of communities detected in recovery times.

| Communities | Members | Central prefectures |
| --- | --- | --- |
| 1-Southwest  (Chengdu) | Chongqing, Chengdu, Luzhou, Guangan, Zunyi, Dazhou, Nanchong, Neijiang, Mianyang, Ziyang, Deyang, Meishan, Suining, Zigong, Leshan, Bazhong, Tongren, Xiangxi Tujia and Miao Autonomous Prefecture, Guangyuan, Enshi Tujia and Miao Autonomous Prefecture, Ya'an, Hanzhong, Zhangjiajie | Chengdu  Chongqing |
| 2-Northeast | Shenyang, Changchun, Harbin, Suihua, Qiqihar, Anshan, Daqing, Tongliao, Siping, Tieling, Jinzhou, Liaoyang, Songyuan, Fuxin, Panjin, Huludao, Xingan League, Jilin, Dalian, Heihe, Fushun, Baicheng, Yingkou, Liaoyuan, Hulunbeir, Tonghua, Benxi, Jiamusi, Dandong, Yichun, Mudanjiang, Yanbian Korean Autonomous Prefecture, Shuangyashan, Baishan, Qitaihe, Hegang, Jixi, Daxing'anling, Chaoyang, Chifeng, Chengde | Shenyang Changchun Harbin |
| 3-Central China | Zhengzhou, Xuzhou, Fuyang, Zhoukou, Suzhou, Shangqiu, Bozhou, Heze, Jining, Zhumadian, Linyi, Xinyang, Kaifeng, Xinxiang, Xuchang, Zaozhuang, Huaibei, Luoyang, Pingdingshan, Luohe, Rizhao, Jiaozuo, Lu'an, Suqian, Lianyungang | Zhengzhou |
| 4-Jing-Jin-Ji | Beijing, Tianjin, Jinan, Shijiazhuang, Handan, Langfang, Liaocheng, Dezhou, Puyang, Cangzhou, Anyang, Xingtai, Yantai, Baoding, Binzhou, Hengshui, Qingdao, Zibo, Weifang, Taian, Tangshan, Weihai, Dongying, Qinhuangdao, Hebi, Jincheng | Beijing Tianjin Shijiazhuang Jinan |
| 5-Yangtze River Delta | Shanghai, Nanjing, Hangzhou, Suzhou, Wuxi, Chuzhou, Changzhou, Jiaxing, Taizhou, Yancheng, Ma’anshan, Nantong, Yangzhou, Zhenjiang, Wuhu, Xuancheng, Shaoxing, Ningbo, Huzhou, Jinhua, Taizhou, Huangshan, Zhoushan, Huaian, Lishui, Hefei, Huainan, Bengbu, Quzhou | Shanghai Nanjing Hangzhou |
| 6-Pearl River Delta | Guangzhou, Shenzhen, Dongguan, Foshan, Zhongshan, Huizhou, Jieyang, Shantou, Zhuhai, Zhaoqing, Jiangmen, Qingyuan, Chaozhou, Yunfu, Shanwei, Meizhou, Heyuan, Heyuan, Nanning, Maoming, Zhanjiang, Yulin, Guigang, Qinzhou, Beihai, Wuzhou, Chongzuo, Fangchenggang, Yangjiang, Hengyang, Shaoyang, Yongzhou, Chenzhou, Shaoguan, Hezhou, Liuzhou, Laibin, Huaihua, Qiandongnan Miao and Dong Autonomous Prefecture | Guangzhou Shenzhen Dongguan Foshan |
| 7-South (Haikou) | Haikou, Sanya, Chengmai, Wenchang, Danzhou, Ding'an, Qionghai, Wanning, Lingshui Li Autonomous County, Lingao, Ledong Li Autonomous County, Dongfang, Tunchang, Changjiang Li Autonomous County, Baisha Li Autonomous County, Baoting Li and Miao Autonomous County, Qiongzhong Li and Miao Autonomous County, Wuzhishan, Sansha | Haikou |
| 8-Northwest  (Urumqi) | Urumqi, Kashgar, Changji Hui Autonomous Prefecture, Kizilsu Kirgiz Autonomous Prefecture, Ili Kazakh Autonomous Prefecture, Bayingolin Mongolian Autonomous Prefecture, Tacheng, Shihezi, Tumushuke, Tiemenguan, Aksu, Aletai, Wujiaqu, Beitun, Karamay, Bortala Mongolian Autonomous Prefecture, Hotan, Turpan, Hami, Alaer, KekeDala, Shuanghe, Kunyu | Urumqi |
| 9-South Central (Wuhan) | Wuhan, Nanchang, Huanggang, Jiujiang, Shangrao, Yichun, Ganzhou, Xiaogan, Ji'an, Huangshi, Ezhou, Jingdezhen, Tianmen, Yingtan, Xinyu, Qianjiang, Anqing, Chizhou, Tongling, Xiamen, Fuzhou, Quanzhou, Zhangzhou, Ningde, Putian, Longyan, Sanming, Nanping, Wenzhou | Wuhan Xiamen |
| 10-Northwest  (Xi’an) | Xi'an, Lanzhou, Xining, Xianyang, Yuncheng, Weinan, Sanmenxia, Haidong, Pingliang, Linfen, Qingyang, Dingxi, Baoji, Linxia Hui Autonomous Prefecture, Zhongwei, Tianshui, Longnan, Guyuan, Baiyin Yanan, Shangluo, Gannan Tibetan Autonomous Prefecture, Wuwei, Tongchuan, Jiuquan, Alxa League, Tibetan Autonomous Prefecture of Huangnan, Jiayuguan, Jinchang, Zhangye, Hainan Tibetan Autonomous Prefecture, Haibei Tibetan Autonomous Prefecture, Haixi Mongolian and Tibetan Autonomous Prefecture, Yushu Tibetan Autonomous Prefecture, Golog Tibetan Autonomous Prefecture, Shennongjia, Nanyang, Shiyan, Ankang, Xiangyang, Suizhou, Ganzi Tibetan Autonomous Prefecture, Aba Tibetan and Qiang Autonomous Prefecture | Xi’an Lanzhou |
| 11-Southwest（Lhasa） | Lhasa, Shannan, Shigatse, Naqu, Linzhi, Ali, Changdu | Lhasa |
| 12-South Central (Changsha) | Changsha, Yueyang, Jingzhou, Changde, Zhuzhou, Xiangtan, Yiyang, Loudi, Pingxiang, Guilin, Xianning, Jingmen, Yichang, Xiantao Tianmen, Qianjiang | Changsha |
| 13-Southwest (Guiyang) | Guiyang, Kunming, Zhaotong, Liangshan Yi Autonomous Prefecture, Yibin, Bijie, Liupanshui, Qujing, Qiannan Buyi and Miao Autonomous Prefecture, Anshun, Qianxinan Buyi and Miao Autonomous Prefecture, Baise, Hechi, Dali Bai Autonomous Prefecture, Panzhihua, Honghe Hani Yi Autonomous Prefecture, Chuxiong Yi Autonomous Prefecture, Lijiang, Yuxi, Wenshan Zhuang and Miao Autonomous Prefecture, Lincang, Baoshan, Pu'er, Dehong Dai Jingpo Autonomous Prefecture, Xishuangbanna Dai Autonomous Prefecture, Diqing Tibetan Autonomous Prefecture, Nujiang Lisu Autonomous Prefecture | Guiyang |
| 14-Northwest（Taiyuan） | Taiyuan, Yinchuan, Yulin, Luliang, Ordos, Xinzhou, Jinzhong, Wuzhong, Xilingole League, Shuozhou, Datong, Hohhot, Baotou, Ulanqab, Bayan Nur, Zhangjiakou, Changzhi, Wuhai, Shizuishan, Yangquan | Taiyuan |

Table S8. List of super-tier and first-tier cities in China, 2020.

| City name | City tier | City name | City tier | City name | City tier |
| --- | --- | --- | --- | --- | --- |
| Beijing | Super tier | Shanghai | Super tier | Guangzhou | Super tier |
| Shenzhen | Super tier | Chengdu | First-tier | Chongqing | First-tier |
| Hangzhou | First-tier | Wuhan | First-tier | Xi’an | First-tier |
| Tianjin | First-tier | Suzhou | First-tier | Nanjing | First-tier |
| Zhengzhou | First-tier | Changsha | First-tier | Dongguan | First-tier |
| Shenyang | First-tier | Qingdao | First-tier | Hefei | First-tier |
| Foshan | First-tier |  |  |  |  |

Table S9. List of the second-tier cities in China, 2020.

| City name | City tier | City name | City tier | City name | City tier |
| --- | --- | --- | --- | --- | --- |
| Ningbo | Second-tier | Kunming | Second-tier | Fuzhou | Second-tier |
| Wuxi | Second-tier | Xiamen | Second-tier | Jinan | Second-tier |
| Dalian | Second-tier | Harbin | Second-tier | Wenzhou | Second-tier |
| Shijiazhuang | Second-tier | Quanzhou | Second-tier | Nanning | Second-tier |
| Changchun | Second-tier | Nanchang | Second-tier | Guiyang | Second-tier |
| Jinhua | Second-tier | Changzhou | Second-tier | Huizhou | Second-tier |
| Jiaxing | Second-tier | Nantong | Second-tier | Xuzhou | Second-tier |
| Taiyuan | Second-tier | Zhuhai | Second-tier | Zhongshan | Second-tier |
| Baoding | Second-tier | Lanzhou | Second-tier | Taizhou | Second-tier |
| Shaoxing | Second-tier | Langfang | Second-tier | Yantai | Second-tier |

Table S10. Lists of the third-tier of cities in China, 2020.

| City name | City tier | City name | City tier | City name | City tier |
| --- | --- | --- | --- | --- | --- |
| Weifang | Third-tier | Yangzhou | Third-tier | Haikou | Third-tier |
| Shantou | Third-tier | Luoyang | Third-tier | Urumqi | Third-tier |
| Linyi | Third-tier | Tangshan | Third-tier | Zhenjiang | Third-tier |
| Yancheng | Third-tier | Huzhou | Third-tier | Ganzhou | Third-tier |
| Taizhou | Third-tier | Jining | Third-tier | Hohhot | Third-tier |
| Xianyang | Third-tier | Zhangzhou | Third-tier | Jieyang | Third-tier |
| Jiangmen | Third-tier | Guilin | Third-tier | Handan | Third-tier |
| Wuhu | Third-tier | Sanya | Third-tier | Fuyang | Third-tier |
| Huaian | Third-tier | Zunyi | Third-tier | Yinchuan | Third-tier |
| Hengyang | Third-tier | Shangrao | Third-tier | Liuzhou | Third-tier |
| Zibo | Third-tier | Putian | Third-tier | Mianyang | Third-tier |
| Zhanjiang | Third-tier | Shangqiu | Third-tier | Xinyang | Third-tier |
| Jiujiang | Third-tier | Xinxiang | Third-tier | Nanyang | Third-tier |
| Lianyungang | Third-tier | Cangzhou | Third-tier | Yichang | Third-tier |
| Zhumadian | Third-tier | Bengbu | Third-tier | Yueyang | Third-tier |
| Xiangyang | Third-tier | Suqian | Third-tier | Weihai | Third-tier |
| Chuzhou | Third-tier | Zhuzhou | Third-tier | Ningde | Third-tier |
| Xingtai | Third-tier | Chaozhou | Third-tier | Qinhuangdao | Third-tier |
| Zhaoqing | Third-tier | Jingzhou | Third-tier | Zhoukou | Third-tier |
| Ma’anshan | Third-tier | Qingyuan | Third-tier | Suzhou | Third-tier |
| Anshan | Third-tier | Anqing | Third-tier | Heze | Third-tier |
| Yichun | Third-tier | Huanggang | Third-tier | Taian | Third-tier |
| Nanchong | Third-tier | Lu’an | Third-tier | Daqing | Third-tier |
| Zhoushan | Third-tier |  |  |  |  |

Table S11. Lists of the fourth-tier of cities in China, 2020.

| City name | City tier | City name | City tier | City name | City tier |
| --- | --- | --- | --- | --- | --- |
| Changde | Fourth-tier | Weinan | Fourth-tier | Xiaogan | Fourth-tier |
| Lishui | Fourth-tier | Yuncheng | Fourth-tier | Dezhou | Fourth-tier |
| Xuchang | Fourth-tier | Xiangtan | Fourth-tier | Jinzhong | Fourth-tier |
| Anyang | Fourth-tier | Sanming | Fourth-tier | Kaifeng | Fourth-tier |
| Chenzhou | Fourth-tier | Maoming | Fourth-tier | Shaoyang | Fourth-tier |
| Deyang | Fourth-tier | Longyan | Fourth-tier | Nanping | Fourth-tier |
| Huainan | Fourth-tier | Huangshi | Fourth-tier | Yingkou | Fourth-tier |
| Rizhao | Fourth-tier | Xining | Fourth-tier | Quzhou | Fourth-tier |
| Dongying | Fourth-tier | Jilin | Fourth-tier | Shaoguan | Fourth-tier |
| Zaozhuang | Fourth-tier | Baotou | Fourth-tier | Xuancheng | Fourth-tier |
| Huaihua | Fourth-tier | Panjin | Fourth-tier | Jinzhou | Fourth-tier |
| Yulin | Fourth-tier | Beihai | Fourth-tier | Baoji | Fourth-tier |
| Fuzhou | Fourth-tier | Jingdezhen | Fourth-tier | Pingdingshan | Fourth-tier |
| Jiaozuo | Fourth-tier | Yibin | Fourth-tier | Xianning | Fourth-tier |
| Shanwei | Fourth-tier | Shiyan | Fourth-tier | Yulin | Fourth-tier |
| Binzhou | Fourth-tier | Ji'an | Fourth-tier | Yongzhou | Fourth-tier |
| Yiyang | Fourth-tier | Qiannan | Fourth-tier | Dandong | Fourth-tier |
| Qujing | Fourth-tier | Leshan | Fourth-tier | Qiandongnan | Fourth-tier |
| Zhangjiakou | Fourth-tier | Huangshan | Fourth-tier | Ordos | Fourth-tier |
| Yangjiang | Fourth-tier | Luzhou | Fourth-tier | Tongling | Fourth-tier |
| Hengshui | Fourth-tier | Enshi | Fourth-tier | Honghe | Fourth-tier |
| Dali | Fourth-tier | Datong | Fourth-tier | Chengde | Fourth-tier |
| Luohe | Fourth-tier | Huludao | Fourth-tier | Heyuan | Fourth-tier |
| Loudi | Fourth-tier | Yanbian | Fourth-tier | Qiqihar | Fourth-tier |
| Yan'an | Fourth-tier | Fushun | Fourth-tier | Lhasa | Fourth-tier |
| Changzhi | Fourth-tier | Dazhou | Fourth-tier | Ezhou | Fourth-tier |
| Xinzhou | Fourth-tier | Lvliang | Fourth-tier | Huaibei | Fourth-tier |
| Puyang | Fourth-tier | Meishan | Fourth-tier | Chizhou | Fourth-tier |
| Jingmen | Fourth-tier | Bozhou | Fourth-tier | Linfen | Fourth-tier |
| Liaocheng | Fourth-tier | Meizhou | Fourth-tier | Tongren | Fourth-tier |

Table S12. Lists of the fifth-tier of cities in China, 2020.

| City name | City tier | City name | City tier | City name | City tier |
| --- | --- | --- | --- | --- | --- |
| Hanzhong | Fifth-tier | Liaoyang | Fifth-tier | Wuzhou | Fifth-tier |
| Yingtan | Fifth-tier | Baise | Fifth-tier | Bijie | Fifth-tier |
| Qinzhou | Fifth-tier | Yunfu | Fifth-tier | Jiamusi | Fifth-tier |
| Chaoyang | Fifth-tier | Guigang | Fifth-tier | Lijiang | Fifth-tier |
| Siping | Fifth-tier | Neijiang | Fifth-tier | Liupanshui | Fifth-tier |
| Anshun | Fifth-tier | Sanmenxia | Fifth-tier | Chifeng | Fifth-tier |
| Xinyu | Fifth-tier | Mudanjiang | Fifth-tier | Jincheng | Fifth-tier |
| Zigong | Fifth-tier | Benxi | Fifth-tier | Fangchenggang | Fifth-tier |
| Tieling | Fifth-tier | Suizhou | Fifth-tier | Guang'an | Fifth-tier |
| Guangyuan | Fifth-tier | Tianshui | Fifth-tier | Suining | Fifth-tier |
| Pingxiang | Fifth-tier | Xishuangbanna | Fifth-tier | Suihua | Fifth-tier |
| Hebi | Fifth-tier | Xiangxi | Fifth-tier | Songyuan | Fifth-tier |
| Fuxin | Fifth-tier | Jiuquan | Fifth-tier | Zhangjiajie | Fifth-tier |
| Guizhou | Fifth-tier | Baoshan | Fifth-tier | Zhaotong | Fifth-tier |
| Karamay | Fifth-tier | Hulun Buir | Fifth-tier | Hezhou | Fifth-tier |
| Tonghua | Fifth-tier | Yangquan | Fifth-tier | Hechi | Fifth-tier |
| Laibin | Fifth-tier | Yuxi | Fifth-tier | Ankang | Fifth-tier |
| Tongliao | Fifth-tier | Dehong | Fifth-tier | Chuxiong | Fifth-tier |
| Shuozhou | Fifth-tier | Yili | Fifth-tier | Wenshan | Fifth-tier |
| Jiayuguan | Fifth-tier | Liangshan | Fifth-tier | Ziyang | Fifth-tier |
| Xilingol | Fifth-tier | Ya'an | Fifth-tier | Pu'er | Fifth-tier |
| Chongzuo | Fifth-tier | Qingyang | Fifth-tier | Bayingolin | Fifth-tier |
| Ulanqab | Fifth-tier | Baishan | Fifth-tier | Changji | Fifth-tier |
| Baicheng | Fifth-tier | Xing’an | Fifth-tier | Dingxi | Fifth-tier |
| Kashi | Fifth-tier | Baiyin | Fifth-tier | Longnan | Fifth-tier |
| Zhangye | Fifth-tier | Shangluo | Fifth-tier | Heihe | Fifth-tier |
| Hami | Fifth-tier | Wuzhong | Fifth-tier | Panzhihua | Fifth-tier |
| Bayan Nur | Fifth-tier | Bazhong | Fifth-tier | Jixi | Fifth-tier |
| Wuhai | Fifth-tier | Lincang | Fifth-tier | Haidong | Fifth-tier |
| Shuangyashan | Fifth-tier | Aksu | Fifth-tier | Shizuishan | Fifth-tier |
| Alashan | Fifth-tier | Haixi | Fifth-tier | Pingliang | Fifth-tier |
| Liaoyuan | Fifth-tier | Linxia | Fifth-tier | Tongchuan | Fifth-tier |
| Jinchang | Fifth-tier | Hegang | Fifth-tier | Yichun | Fifth-tier |
| Linzhi | Fifth-tier | Guyuan | Fifth-tier | Wuwei | Fifth-tier |
| Danzhou | Fifth-tier | Turpan | Fifth-tier | Ganzi | Fifth-tier |
| Zhongwei | Fifth-tier | Nujiang | Fifth-tier | Hetian | Fifth-tier |
| Diqing | Fifth-tier | Gannan | Fifth-tier | Ngawa | Fifth-tier |
| Greater Khingan Range | Fifth-tier | Qitaihe | Fifth-tier | Shannan | Fifth-tier |
| Shigatse | Fifth-tier | Tarbagatay | Fifth-tier | Bortala | Fifth-tier |
| Qamdo | Fifth-tier | Altay | Fifth-tier | Yushu | Fifth-tier |
| Hainan | Fifth-tier | Kizilsu | Fifth-tier | Ali | Fifth-tier |
| Haibei | Fifth-tier | Huangnan | Fifth-tier | Golog | Fifth-tier |
| Naqu | Fifth-tier | Sansha | Fifth-tier |  |  |

1. Number of mobile cell phone subscriptions in China from September November 2019 to November. China: mobile phone subscriptions by month 2019-2020, 2021. https://www.statista.com/statistics/278204/china-mobile-users-by-month/ [↑](#footnote-ref-1)
2. National Bureau of Statistics of China. http://www.stats.gov.cn [↑](#footnote-ref-2)
3. https://qianxi.baidu.com/ [↑](#footnote-ref-3)
4. Ministry of Industry and Information Technology of the People's Republic of China.

   *Regulations on the Protecting the personal information of Telecommunications and Internet Users.* http://www.miit.gov.cn/n1146285/n1146352/n3054355/n3057724/n3057729/c470

   0145/content.html. [↑](#footnote-ref-4)
5. GSMA . *GSMA Guidelines on the Protection of Privacy in the Use of mobile phone data for Responding to the Ebola Outbreak.*

   https://www.gsma.com/mobilefordevelopment/resources/gsma-guidelines-on-theprotection-

   of-privacy-in-the-use-of-mobile-phone-data-for-responding-to-the-ebola outbreak. [↑](#footnote-ref-5)
6. Chen Y. The distance-decay function of geographical gravity model: power law or exponential law? *Chaos Solitons Fractals* 2015; **77**: 174–89. [↑](#footnote-ref-6)
7. Liu E and Xiao-Yong Y. New parameter-free mobility model: opportunity priority selection model. *Physica A* 2019; **526**: 121023. [↑](#footnote-ref-7)
8. Liu EJ and Yan XY. A universal opportunity model for human mobility. *Sci Rep* 2020; **10**: 4657. [↑](#footnote-ref-8)
9. Sallah K, Giorgi R and Bengtsson L *et al*. Mathematical models for predicting human mobility in the context of infectious disease spread: introducing the impedance model. *Int J Health Geogr* 2017; **16**: 42. [↑](#footnote-ref-9)
